# Supplementary material for: Cell-cycle exit and stem cell differentiation are coupled through regulation of mitochondrial activity in the Drosophila testis
Source: Cell Rep. 2022 May 10;39(6):110774. doi: 10.1016/j.celrep.2022.110774 (PMC9350557; doi:10.1016/j.celrep.2022.110774)
Supplement: Document S1. Figures S1–S7 and Table S1 [file mmc1.pdf]

Cell Reports, Volume *39*

Supplemental information

Cell-cycle exit and stem cell differentiation are coupled through regulation of mitochondrial activity in the *Drosophila* testis

Diego Sainz de la Maza, Silvana Hof-Michel, Lee Phillimore, Christian Bökel, and Marc Amoyel

Table S1. Clone recovery rates (% testes) for the indicated genotypes. Related to Figures 1, 2 and S2.

Clone recovery rates that are significantly different from their respective controls are indicated by blue shading. All others are not significantly different, as determined by Fisher's exact test.

N.B. the *Cdk4*^{s4639} experiment was carried out separately from other FRT^{42D} experiments with an independent control.

ND: Not determined.

**Genotype**	**2 dpci (n)**	**7 dpci (n)**	**14 dpci (n)**
Control (*FRT*^{40A})	43 (23)	48 (54)	ND
CycE^{AR95}	16 (31)	0 (37)	ND
CycE^{WX}	60.5 (43)	29.3 (75)	ND
Control (*FRT*^{42D})	67 (95)	55 (148)	65 (46)
Dp^{a3}	67 (18)	56 (55)	66 (29)
Dp^{a4}	ND	62 (47)	67 (30)
Cdk4^{k06503}	53 (38)	47 (34)	ND
Control (*FRT*^{42D})	67 (42)	50 (42)	ND
Cdk4^{s4639}	ND	33 (52)	ND
Control (*FRT*^{82B})	42 (45)	45 (93)	ND
E2f1^{rM729}	45 (33)	33 (55)	ND

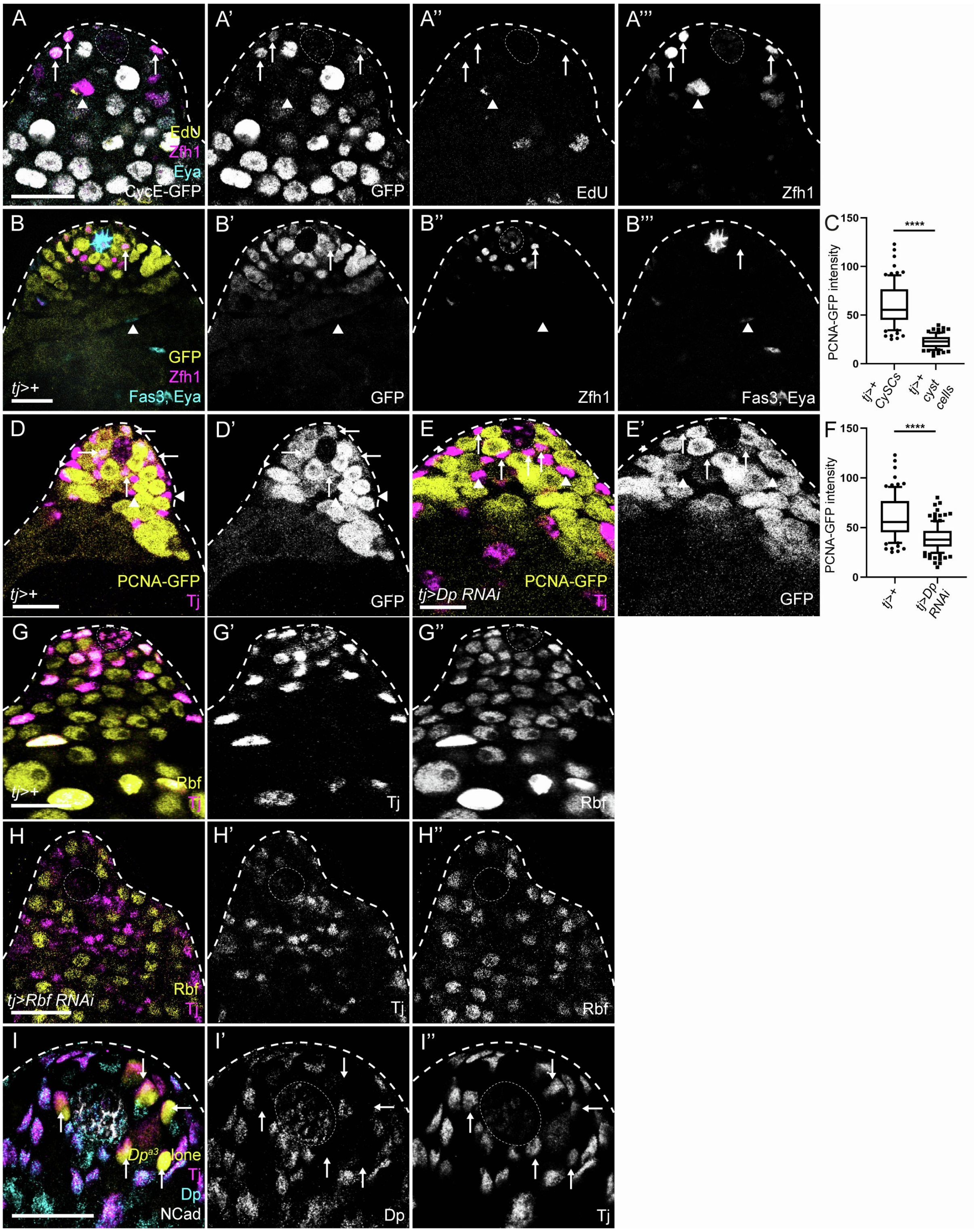

Figure S1. Expression and endogenous activity of G1/S regulators in the *Drosophila* testis. Related to Figures 1 and 2. (A) Testis from a CycE protein trap (CycE-GFP) line, labelled with EdU (yellow, A") and antibodies against GFP (white, A'), Zfh1 (magenta, A''') to label CySCs and Eya (cyan) to label cyst cells. GFP expression is detected in a subset of CySCs (arrows), but absent from CySCs that are in S-phase, identified by EdU incorporation (arrowhead). (B) Expression of the canonical E2f1/Dp reporter *PCNA-GFP* (yellow, single channel in B') in the testis. Zfh1 (magenta, B') labels CySCs and

their immediate daughters. Eya labels differentiated cyst cells (cyan, B”’). *PCNA-GFP* is present in a subset of CySCs (arrow), consistent with a cyclic pattern of expression of PCNA. No signal can be observed in Eya-positive cyst cells (arrowhead). (C) Quantification of PCNA-GFP signal in CySCs and differentiated cyst cells, showing a significant difference in GFP intensity. **** denotes P<0.0001, Mann-Whitney test. (D-F) Knockdown of Dp decreases PCNA-GFP signal. Tj (magenta) labels CySCs and early cyst cells. In controls (D), GFP expression is detected in Tj-positive CySCs adjacent to the hub (arrows), while no expression is present in differentiated cyst cells (arrowhead). (E) Dp knockdown in the somatic lineage results in decreased expression in Tj-positive cells around the hub (arrows), indicating that PCNA-GFP expression reports on endogenous E2f1/Dp activity. (F) Quantification of PCNA-GFP intensity in CySCs adjacent to the hub in controls and Dp knockdowns. **** denotes P<0.0001, Mann-Whitney test. (G-H) Testes labelled with antibodies against Rbf (yellow, single channels in G”,H”) and Tj (magenta, single channels in G’,H’). In controls (G), Rbf is detected in all cells in the Drosophila testis, including CySCs adjacent to the hub (dotted line) and differentiating cyst cells. Expression increases in cyst cells distant from the hub. Rbf is also present in germ cells and weakly in hub cells. (H) Expression of Rbf RNAi with *tj-Gal4* results in a complete lack of any detectable Rbf protein in Tj-positive cells. (I) Positively-labelled clones mutant for *Dp* labelled with antibodies against Dp (cyan, I’), Tj (magenta, I”), GFP to mark the clone (yellow) and NCad to mark the hub (white). Mutant clones lack Dp protein (arrows). Dotted lines outline the hub. Scale bars: 20μm.

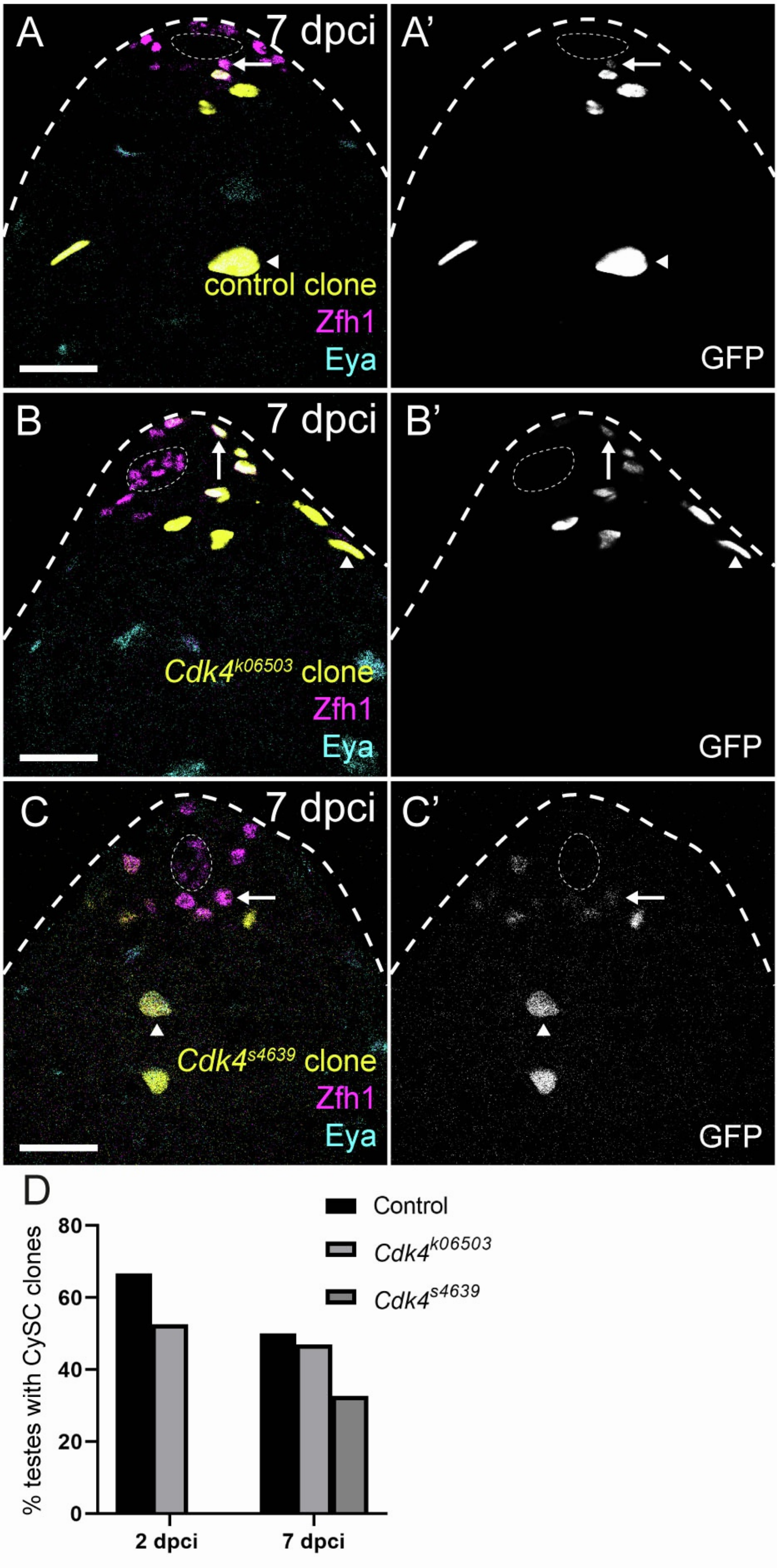

A
7 dpci
control clone
Zfh1
Eya
A'
GFP
B
7 dpci
Cdk4^{k06503} clone
Zfh1
Eya
B'
GFP
C
7 dpci
Cdk4^{s4639} clone
Zfh1
Eya
C'
GFP
D
Control
Cdk4^{k06503}
Cdk4^{s4639}
% testes with CySC clones
80
60
40
20
0
2 dpci
7 dpci

Figure S2. *Cdk4* is not required for CySC self-renewal. Related to Figure 2. Positively-marked clones labelled with GFP expression (yellow, single channels in A',B',C') were generated and recovered at 7 days post clone induction (dpci). Zfh1 (magenta) identifies CySCs and early daughters, and Eya (cyan) marks differentiated cyst cells. Control clones (A) at 7 dpci contained both Zfh1-expressing CySCs (arrow) and Eya-positive cyst cells (arrowhead). Clones mutant for independent alleles of *cdk4* (B,C) were similarly recovered at 7 dpci and contained Zfh1-expressing CySCs (arrows), indicating that *Cdk4* is not required for CySC self-renewal. (D) Quantification of clone recovery rates, showing the fraction of testes containing controls and *Cdk4* mutant CySC clones. No significant differences were observed between the recovery rates of control and mutant clones, as determined by Fisher's exact test. See Table S1 for n values. Dotted lines outline the hub. Scale bars: 20μm.

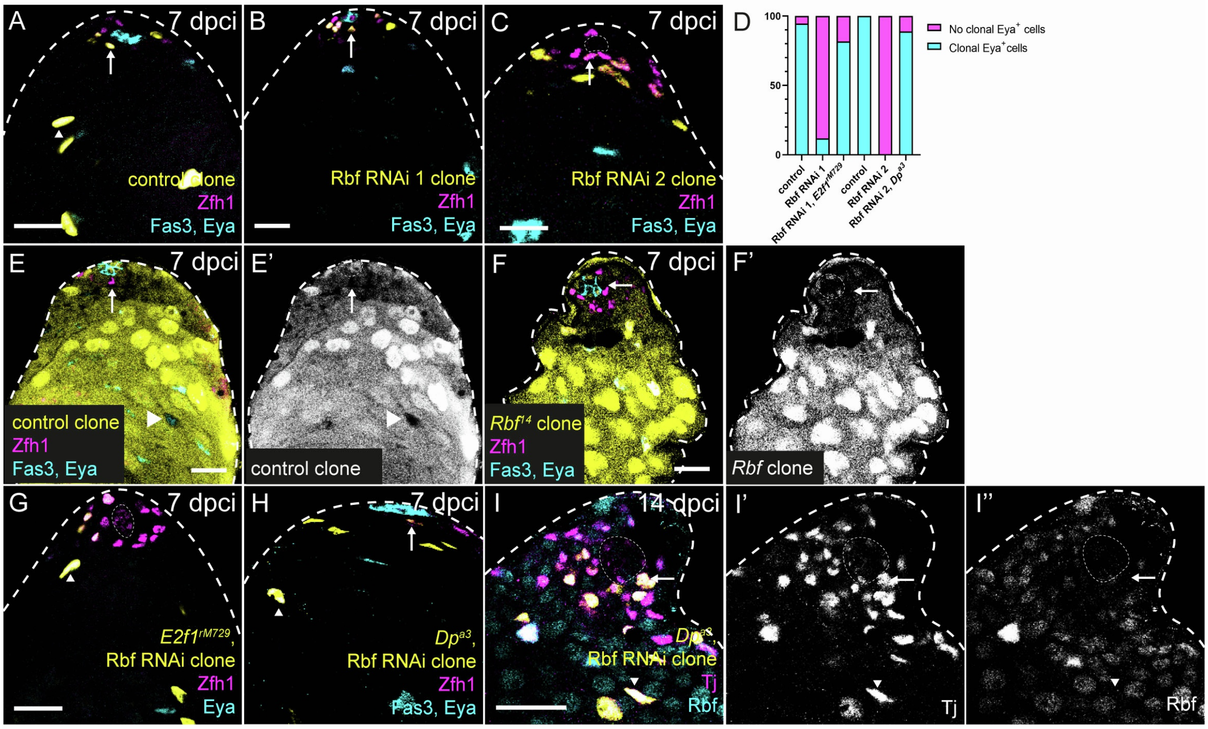

Figure S3. Clonal loss-of-function of *Rbf* recapitulates the phenotype of lineage-wide knockdown. Related to Figure 3. (A-C) Positively-marked CySC clones expressing GFP (yellow). Zfh1 (magenta) marks CySCs and early daughters, Eya (cyan) marks differentiated cyst cells and Fas3 marks the hub. Control clones at 7 dpci (A) were recovered adjacent to the hub (arrow) and contained differentiated cyst cells (arrowhead). (B,C) Clones expressing two independent RNAi constructs targeting Rbf consisted exclusively of Zfh1-expressing cells and contained no Eya-expressing cyst cells. (D) Frequency plot showing the proportion of clones containing Eya-positive cells in the different genotypes. (E,F) Control (E) or *Rbf* mutant (F) negatively-marked clones, identified by lack of GFP expression (yellow, single channel in E',F'). Zfh1 (magenta) labels CySCs, Eya (cyan) marks differentiated cyst cells and Fas3 (cyan) marks the hub. See Methods for information about genotypes. Control clones were composed of both Zfh1-positive CySCs (arrow) and Eya-positive cyst cells (arrowhead) at 7 dpci. (F) In contrast, *Rbf* hemizygous mutant clones contained only Zfh1-expressing cells (arrow), indicating that the *Rbf* mutant phenotype in CySCs is similar to RNAi knockdown. (G-I) Differentiation of Rbf RNAi clones is rescued by loss-of-function of *E2f1* or *Dp*. Clones mutant for *E2f1* (G) or *Dp* (H) in which Rbf RNAi was expressed were composed of both Zfh1-expressing CySCs (arrows) and Eya-positive differentiated cyst cells (arrowheads). (I) Rbf protein (cyan, single channel in I'') is absent from *Dp* mutant clones expressing Rbf RNAi (yellow). Cyst lineage clones are identified by GFP expression and Tj expression (magenta, single channel I'). Dotted lines outline the hub. Scale bars: 20μm.

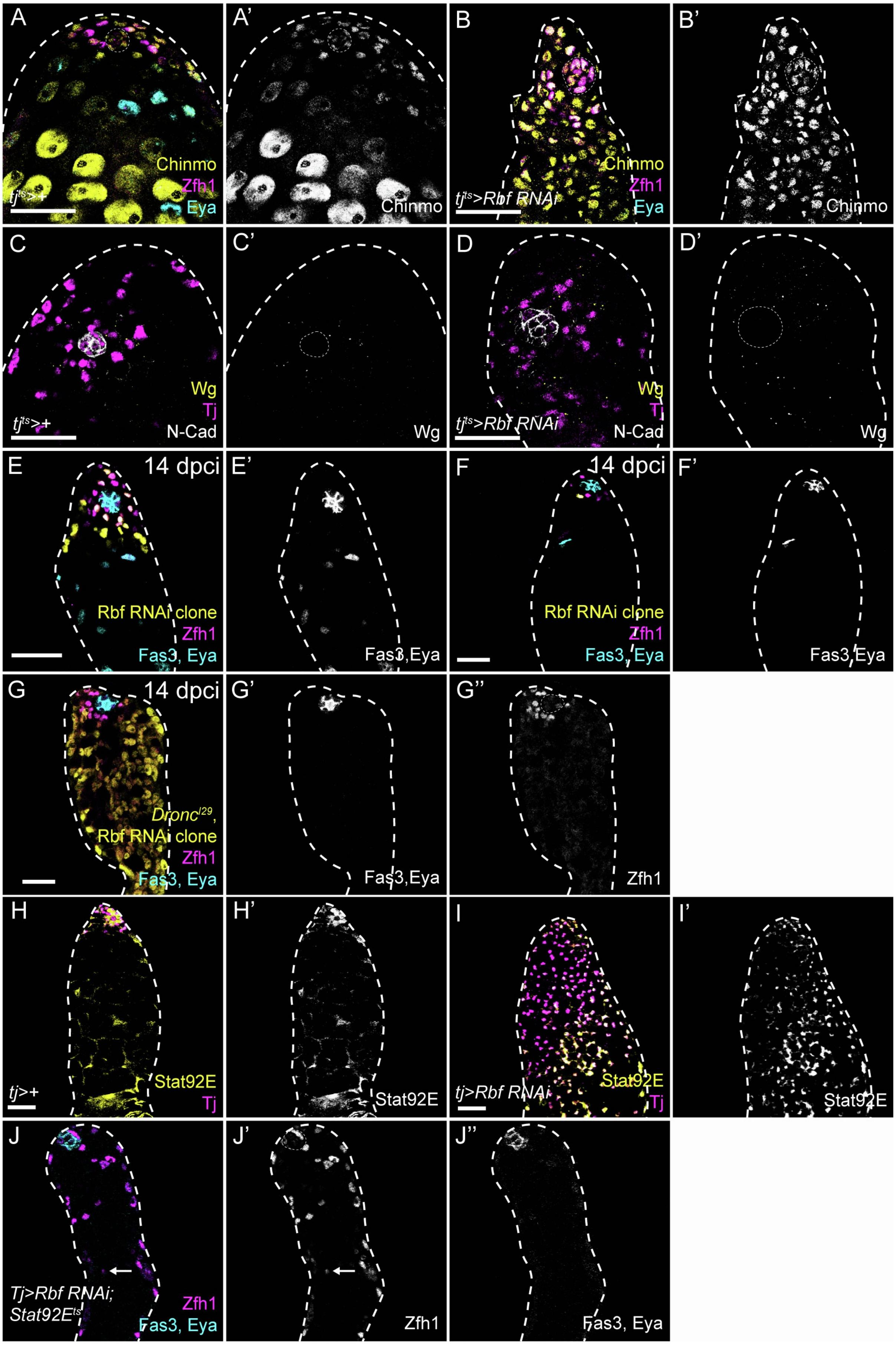

A
A'
B
B'
Chinmo
Zfh1
Eya
tj^ts>+
Chinmo
Chinmo
Zfh1
Eya
tj^ts>Rbf RNAi
Chinmo
C
C'
D
D'
Wg
Tj
N-Cad
tj^ts>+
Wg
Wg
Tj
N-Cad
tj^ts>Rbf RNAi
Wg
E
14 dpci
E'
F
14 dpci
F'
Rbf RNAi clone
Zfh1
Fas3, Eya
Fas3,Eya
Rbf RNAi clone
Zfh1
Fas3, Eya
Fas3,Eya
G
14 dpci
G'
G''
Dronc^I29,
Rbf RNAi clone
Zfh1
Fas3, Eya
Fas3,Eya
Zfh1
H
H'
I
I'
Stat92E
Tj
tj>+
Stat92E
tj>Rbf RNAi
Stat92E
Tj
Stat92E
J
J'
J''
Tj>Rbf RNAi;
Stat92E^ts
Zfh1
Fas3, Eya
Zfh1
Fas3, Eya

Figure S4. Characterisation of ectopic CySCs lacking Rbf. Related to Figure 3. (A-D) Rbf-deficient cells express markers of CySCs. (A,B) Testes labelled with antibodies against Chinmo (yellow, single channel in A’,B’), Zfh1 (magenta) to mark CySCs and early daughters, and Eya (cyan), to mark differentiated cyst cells. In controls (A), Chinmo is expressed in the hub, Zfh1-positive CySCs, GSCs, identified as cells adjacent to the hub negative for Zfh1 expression, and late germ cells. Expression is absent from Eya-positive cyst cells. When Rbf was knocked down in somatic cells with tj^{ts} (B), Chinmo expression was detected in Zfh1-positive somatic cells distant from the hub. Expression of Chinmo in unlabelled early germ cells was also detected throughout the testes. (C,D) Wg expression (yellow, single channels in C’,D’) in testes labelled with antibodies against N-Cadherin (N-Cad, grey) to label the hub and Tj (magenta) to label CySCs and early cyst cells. In controls (C), Wg puncta are visible around somatic cells close to the hub. (D) Wg staining was detected far from the hub in testes with somatic Rbf knockdown indicating an expansion of CySC-like cells. (E-G) CySC clones at 14 dpci positively-labelled with GFP expression. Fas3 (cyan, single channel in E’,F’,G’) labels the hub, Zfh1 (magenta) marks CySCs and early daughter cells, and Eya (cyan) marks differentiated cyst cells. Expression of Rbf RNAi in otherwise wild type clones (E,F) resulted in clones composed only of Zfh1-positive cells and lacking Eya-positive differentiated cyst cells. While most clones contained many cells (E), indicative of proliferation, occasional clones were observed that were composed of very few cells (F). (G) Rbf RNAi expression in clones mutant for the initiator caspase *Dronc* resulted in large clones but these were still devoid of Eya-expressing cyst cells, indicating that cell death restricts the over-proliferation of Rbf-deficient cells but is not responsible for their inability to differentiate. (H-J) Increased JAK/STAT signalling does not mediate the expansion of CySC-like cells caused by Rbf knockdown. (H,I) Testes labelled with antibodies against Stat92E (yellow, single channels in H’,I’), which is stabilised where JAK/STAT signalling is active, and Tj (magenta) to mark CySCs and early cyst cells. In controls (H), Stat92E is detected around the hub. In testes somatically depleted for Rbf (I), stabilised Stat92E is present in Tj-positive cells throughout the testes. (J) Rbf knockdown in the somatic lineage in a temperature-sensitive *Stat92E* mutant (*Stat92E*^{ts}) raised for 10 days at the restrictive temperature results in expansion of Zfh1 (magenta, single channel in J’) away from the hub (arrow) and a lack of Eya-positive cells (cyan, single channel in J”), similar to Rbf knockdown in control animals. Dotted lines outline the hub. Scale bars: 20μm.

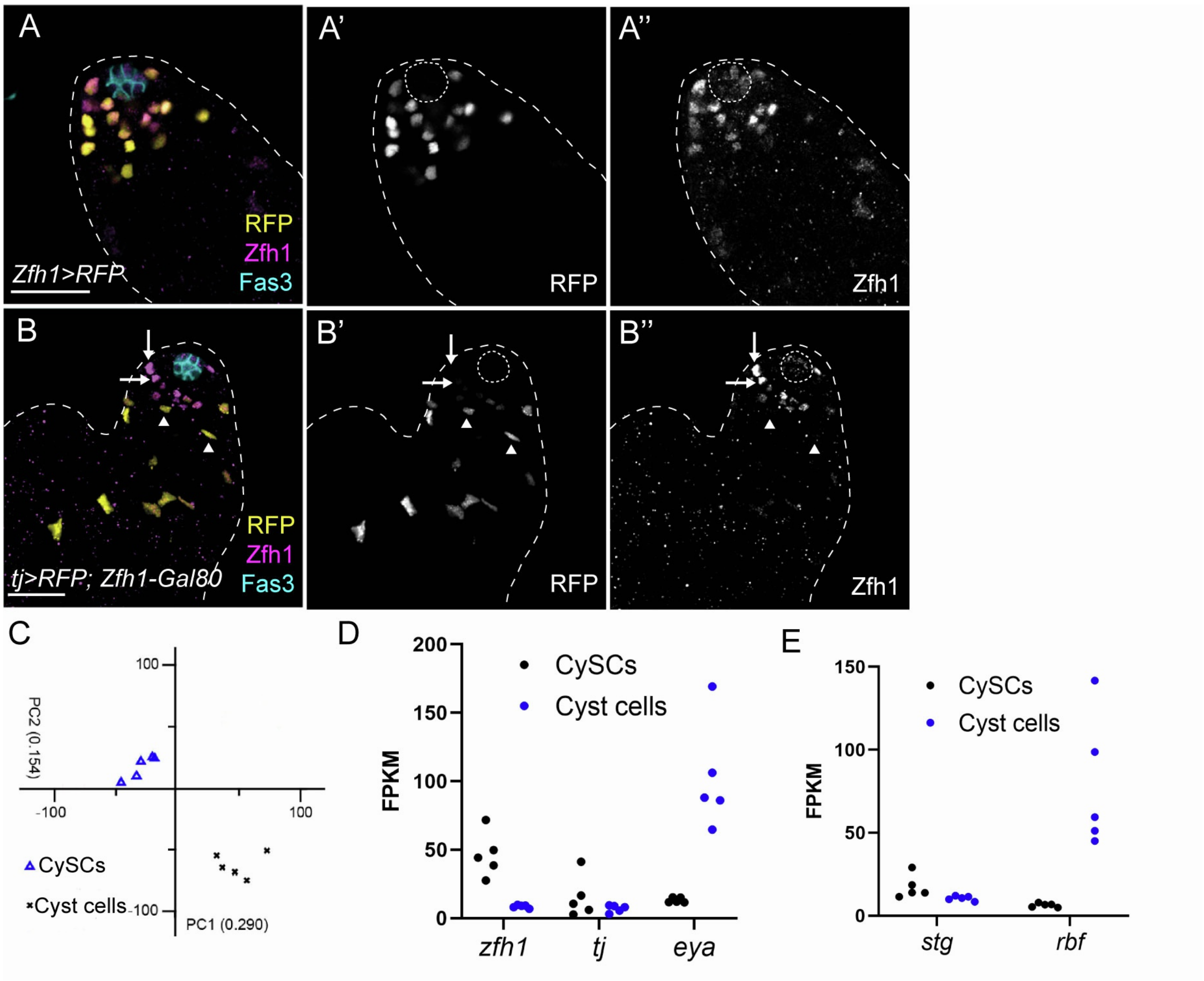

Figure S5. Effective isolation and transcriptome determination of CySCs and cyst cells. Related to Figure 6. (A) Expression of RFP (yellow, single channel A') in *Zfh1>RFP* testes to isolate CySCs. Presence of RFP correlates with Zfh1 expression (magenta, single channel A"). The hub is labelled with Fas3 (cyan). (B) Expression of RFP (yellow, single channel in B') in *tj>RFP; Zfh1-Gal80* testes to isolate differentiating cyst cells. Zfh1-positive cells (magenta, single channel in B") close to the hub (Fas3, cyan) do not express RFP (arrows), whereas early differentiating cells (arrowheads) negative for Zfh1 express RFP. Dotted lines outline the hub. Scale bars: 20μm. (C) Principal component analysis of the transcriptomes of five biological replicates of sorted cell populations showing that CySCs and cyst cells cluster separately. (D) Individual values for reads (in Fragments Per Kilobase of transcript per Million mapped reads (FPKM)) for each replicate of genes encoding known markers of CySCs and cyst cells in the sorted CySC population (black) and cyst cells (blue). As expected, CySCs express higher levels of *zfh1* and *tj*, whereas early cyst cells express higher levels of *eya*. (E) Individual values for reads in FPKM of genes encoding cell cycle components. *Stg* expression which was enriched in CySCs (black) relative to cyst cells (blue), while *Rbf* was upregulated in cyst cells.

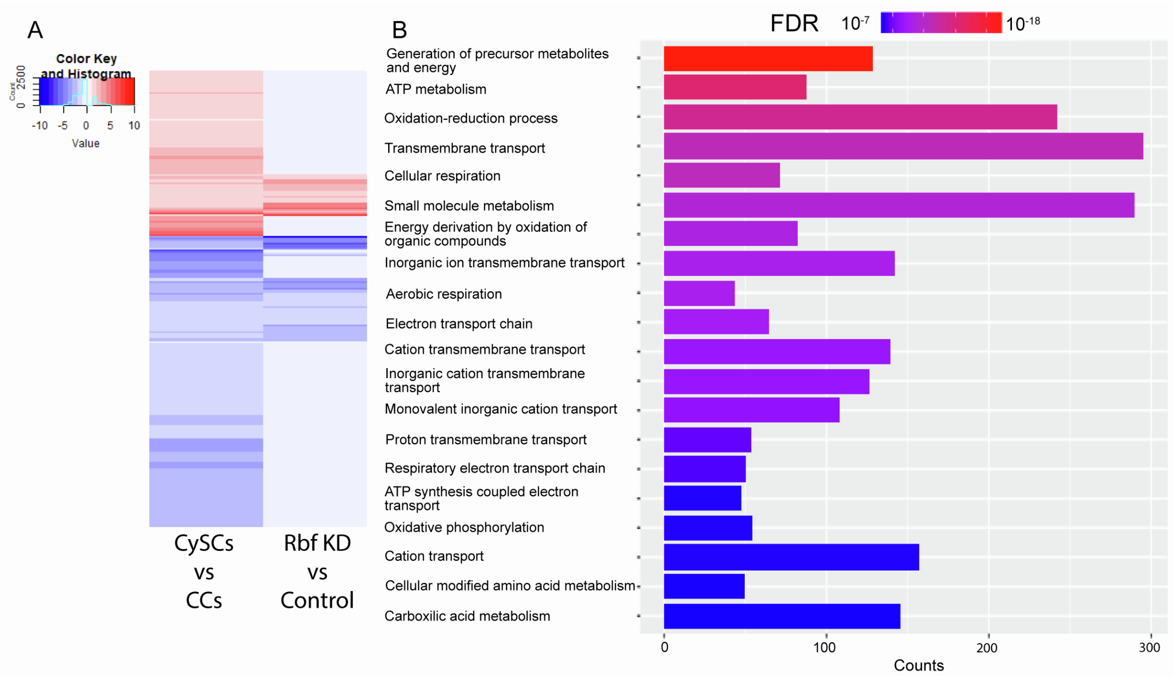

Figure S6. Overlap between the enriched transcripts in CySCs relative to cyst cells and Rbf-deficient CySCs relative to control. Related to Figure 6. (A) Heatmap representing the shared gene differential expression among experiments. Differentially-expressed genes between CySCs and cyst cells (left column) were used as a reference. Genes more highly expressed in CySCs relative to cyst cells are shown in red and genes enriched in cyst cells are shown in blue. Changes for these genes in Rbf knockdown compared to controls are shown in the right column and colour-coded according to differential expression in Rbf knockdowns. Pale blue in the right column indicates genes differentially expressed between control CySCs and cyst cells that were not differentially expressed when Rbf-knockdown somatic cells were compared to controls. Approximately 27% of genes are differentially expressed in both experiments, and show similar valence of expression change. (B) Gene Ontology analysis of genes downregulated in both control CySCs compared to cyst cells and Rbf-deficient cells compared to control. The x axis represents the number of genes in each biological process. Colours of the bars represent the FDR of each category. Shared biological processes include oxidation-reduction processes and many categories related to ATP metabolism, suggesting that somatic knockdown of Rbf results in a similar metabolic gene expression profile to CySCs.

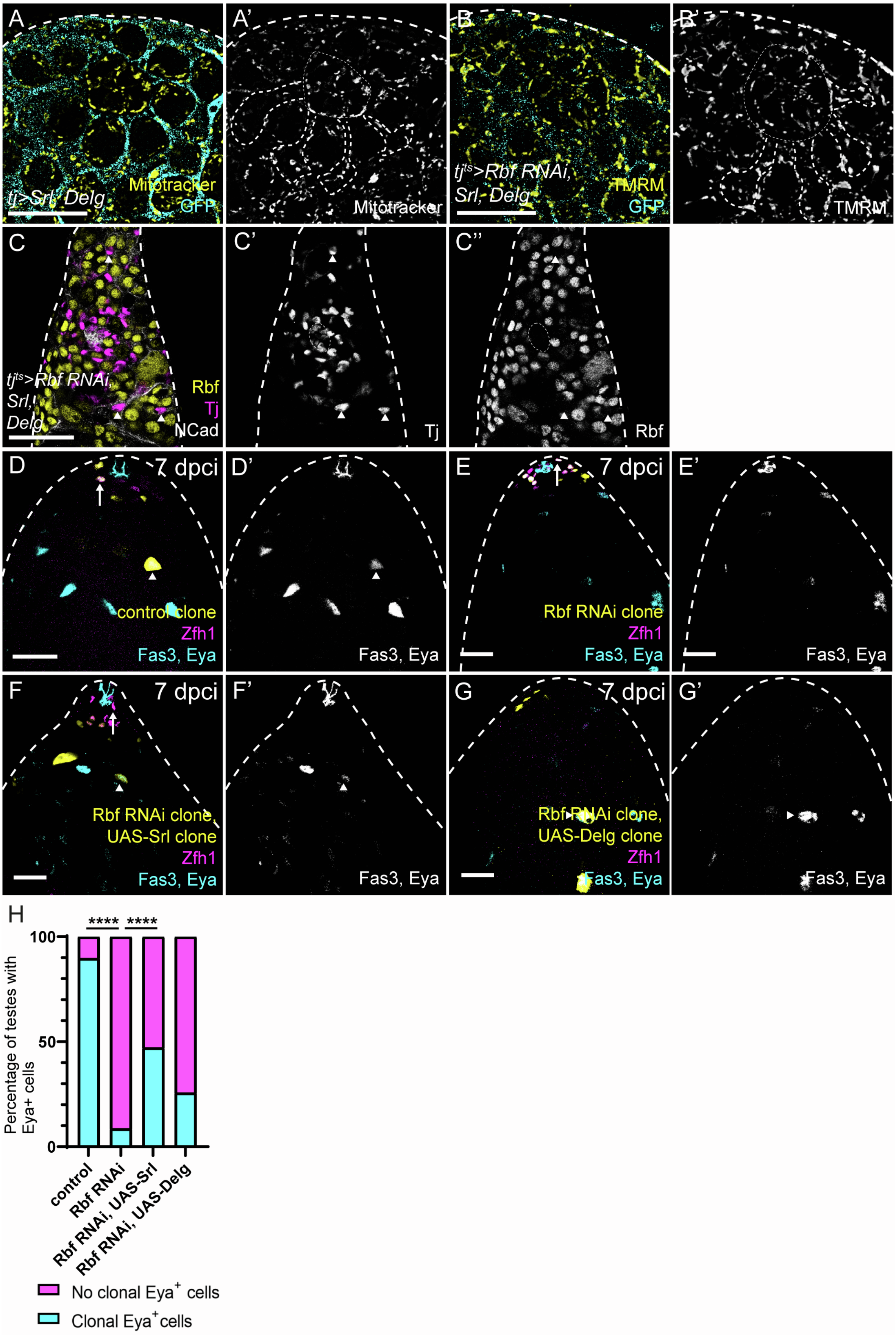

A
tj>Srl, Delg
Mitotracker
GFP
A'
Mitotracker
B
tj^ts>Rbf RNAi, Srl, Delg
TMRM
GFP
B'
TMRM
C
tj^ts>Rbf RNAi, Srl, Delg
Rbf
Tj
NCad
C'
Tj
C''
Rbf
D
7 dpci
control clone
Zfh1
Fas3, Eya
D'
Fas3, Eya
E
7 dpci
Rbf RNAi clone
Zfh1
Fas3, Eya
E'
Fas3, Eya
F
7 dpci
Rbf RNAi clone, UAS-Srl clone
Zfh1
Fas3, Eya
F'
Fas3, Eya
G
7 dpci
Rbf RNAi clone, UAS-Delg clone
Zfh1
Fas3, Eya
G'
Fas3, Eya
H
****
****
Percentage of testes with Eya+ cells
100
50
0
control
Rbf RNAi
Rbf RNAi, UAS-Srl
Rbf RNAi, UAS-Delg
No clonal Eya+ cells
Clonal Eya+ cells

Figure S7. Overexpression of Srl and Delg increases mitochondrial activity and rescues differentiation in Rbf knockdown testes. Related to Figure 7. (A) Testes co-expressing GFP (cyan) in the cyst lineage together with Srl and Delg, labelled with the active mitochondrial dye Mitotracker Red (yellow, single channel in A') and imaged live. Individual CySCs are outlined with a dashed line in A' and the hub is outlined with a dotted line. (B) Testes co-expressing GFP (cyan) in the cyst lineage together with Rbf RNAi, Srl and Delg, labelled with the active mitochondrial dye TMRM (yellow, single channel in B') and imaged live. Individual CySCs are outlined with a dashed line in B' and the hub is outlined with a dotted line. (C) Rbf protein (yellow, C'') is absent in the somatic lineage in testes expressing Rbf RNAi together with Srl and Delg, even in somatic cells away from the hub (arrowheads), indicating that any rescue of differentiation in this background is not due to incomplete knockdown. (D-H) Differentiation is partly rescued by co-expression of Srl or Delg upon clonal loss of Rbf. (D-G) Positively-labelled clones marked by GFP expression (yellow). Zfh1 (magenta) marks CySCs and early daughters, while Eya (cyan, single channel in D',E',F',G') marks differentiated cyst cells. The hub is labelled with Fas3 (cyan). Control clones (D) contained both Zfh1-positive CySCs (arrow) and Eya-positive cyst cells (arrowhead), while only Zfh1-expressing cells were detected in Rbf RNAi-expressing clones (E, arrow). Co-expression of either Srl (F) or Delg (G) with Rbf RNAi resulted in clones that contained Eya-positive cells (arrowheads). In G, the hub is in an adjacent plane. (H) Graph showing the frequency of clones containing Eya-positive cells (cyan bars). Only 9% of Rbf RNAi-expressing clones contained Eya-positive cells ($n=49$ for control, $n=46$ for Rbf RNAi, $P<0.0001$ compared to control clones, Fisher's exact test). Co-expression of Srl resulted in a significant rescue compared to Rbf RNAi alone to 47% ($n=36$, $P<0.0001$), while 26% of clones co-expressing Delg contained Eya-positive cells, approaching statistical significance ($n=35$, $P=0.06$). Dotted lines outline the hub. Scale bars: 20μm.
