# Supplementary material for: Cell-cycle exit and stem cell differentiation are coupled through regulation of mitochondrial activity in the Drosophila testis
Source: Cell Rep. 2022 May 10;39(6):110774. doi: 10.1016/j.celrep.2022.110774 (PMC9350557; doi:10.1016/j.celrep.2022.110774)
Supplement: Document S2. Article plus supplemental information [file mmc3.pdf]

Cell Reports

Article

Cell-cycle exit and stem cell differentiation are coupled through regulation of mitochondrial activity in the *Drosophila* testis

Graphical abstract

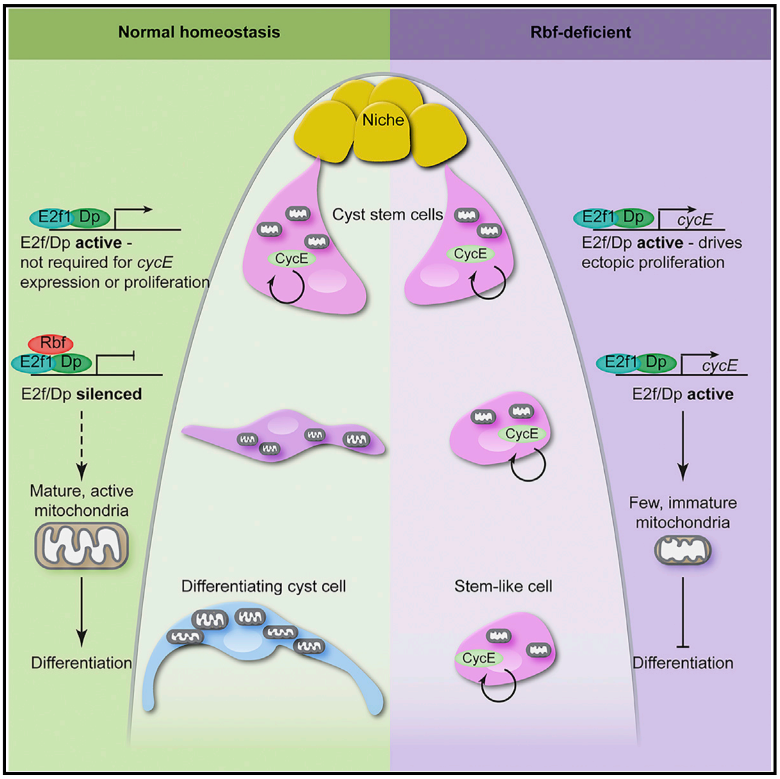

Highlights

- CycE is critical for CySC self-renewal
- E2f/Dp does not act in self-renewal but must be silenced for differentiation
- E2f/Dp inhibits increases in oxidative metabolism involved in normal differentiation
- Increased mitochondrial biogenesis rescues differentiation of E2f/Dp-active cells

Authors

Diego Sainz de la Maza, Silvana Hof-Michel, Lee Phillimore, Christian Bökel, Marc Amoyel

Correspondence

christian.boekel@uni-ulm.de (C.B.), marc.amoyel@ucl.ac.uk (M.A.)

In brief

Stem cell differentiation is linked to their exit from the cell cycle. Here, Sainz de la Maza et al. identify the E2f/Dp transcription factor as an essential link between cell-cycle progression and stem cell identity. They show that E2f/Dp downregulates mitochondrial activity to inhibit differentiation in cycling cells.

Sainz de la Maza et al., 2022, Cell Reports *39*, 110774
May 10, 2022 © 2022 The Author(s).
https://doi.org/10.1016/j.celrep.2022.110774

CellPress

Cell Reports

CellPress
OPEN ACCESS

Article

Cell-cycle exit and stem cell differentiation are coupled through regulation of mitochondrial activity in the *Drosophila* testis

Diego Sainz de la Maza,[1] Silvana Hof-Michel,[2] Lee Phillimore,[1] Christian Bökel,[2,3,*] and Marc Amoyel[1,4,*]
[1]Department of Cell and Developmental Biology, University College London, Gower Street, London WC1E 6BT, UK
[2]Department of Developmental Genetics, Philipps University Marburg, Karl-von-Frisch-Str. 8, 35043 Marburg, Germany
[3]Present address: Core Facility Confocal and Multiphoton Microscopy, Ulm University, Albert-Einstein-Allee 11, 89081 Ulm, Germany
[4]Lead contact
*Correspondence: christian.boekel@uni-ulm.de (C.B.), marc.amoyel@ucl.ac.uk (M.A.)
https://doi.org/10.1016/j.celrep.2022.110774

SUMMARY

Whereas stem and progenitor cells proliferate to maintain tissue homeostasis, fully differentiated cells exit the cell cycle. How cell identity and cell-cycle state are coordinated during differentiation is still poorly understood. The *Drosophila* testis niche supports germline stem cells and somatic cyst stem cells (CySCs). CySCs give rise to post-mitotic cyst cells, providing a tractable model to study the links between stem cell identity and proliferation. We show that, while cell-cycle progression is required for CySC self-renewal, the E2f1/Dp transcription factor is dispensable for self-renewal but instead must be silenced by the *Drosophila* retinoblastoma homolog, Rbf, to permit differentiation. Continued E2f1/Dp activity inhibits the expression of genes important for mitochondrial activity. Furthermore, promoting mitochondrial biogenesis rescues the differentiation of CySCs with ectopic E2f1/Dp activity but not their cell-cycle exit. In sum, E2f1/Dp coordinates cell-cycle progression with stem cell identity by regulating the metabolic state of CySCs.

INTRODUCTION

Adult stem cells maintain tissue homeostasis by balancing self-renewal and differentiation (Jones and Wagers, 2008). In most adult tissues, proliferative capacity is limited to self-renewing stem cells and progenitors or transit-amplifying cells, but terminally differentiated cells are post-mitotic (Ruijtenberg and van den Heuvel, 2016). How cell-cycle state and cell identity are coordinated is still poorly understood.

During development, terminal differentiation is accompanied by permanent cell-cycle exit, with cells usually differentiating in G1, suggesting that the initiation of DNA replication is an important regulated step in ensuring appropriate cell-cycle exit (Buttitta and Edgar, 2007; Ruijtenberg and van den Heuvel, 2016; Soufi and Dalton, 2016).

Progression through the cell cycle is driven by cyclin-dependent kinases (Cdks). Cdk4/6, together with Cyclin D (CycD), are active in the early G1, leading to mono-phosphorylation of the retinoblastoma (Rb) protein. Rb binds a transcription factor, composed of a dimer of transcriptional activator E2f proteins with dimerization partner (Dp), and represses transcription. The steps leading to the inactivation of Rb are still poorly understood (Narasimha et al., 2014; Pennycook and Barr, 2020; Rubin et al., 2020; Soufi and Dalton, 2016); yet, when this inhibition is relieved, E2f/Dp drive transcription of S phase genes and of *Cyclin E* (*CycE*) (Figure 1A). In turn CycE, together with Cdk2, inhibits Rb through further phosphorylation, leading to positive feedback on CycE levels and irreversible entry into S phase (Cappell et al., 2016; Pennycook and Barr, 2020; Rubin et al., 2020; Schwarz et al., 2018).

Cell-cycle regulators play different roles in stem cell fate regulation, depending on the context. Many adult stem cells, such as hematopoietic stem cells, are quiescent; in these, inducing ectopic proliferation results in loss of self-renewal capacity (Cheng et al., 2000). Conversely, in highly proliferative stem cells, such as those residing in *Drosophila* ovaries and testes, mutation of cyclins, Cdks, and the Cdk activator Cdc25, required for cell-cycle progression, results in loss of stem cell maintenance (Ables and Drummond-Barbosa, 2013; Inaba et al., 2011; Wang and Lin, 2005; Wang and Kalderon, 2009), suggesting that cell-cycle progression promotes stem cell maintenance. Consistently, loss of the cell-cycle inhibitor Rb results in expansion of the stem cell and progenitor populations and a block in terminal differentiation (Sage, 2012). Whether these functions of cell-cycle regulators in self-renewal and differentiation are related to their roles in promoting cell-cycle progression, or, as suggested for CycE (Ables and Drummond-Barbosa, 2013), whether they control identity and cell-cycle progression independently remains unclear.

To gain insight into the mechanisms linking cell proliferation and identity in stem cells, we use the *Drosophila* testis as a model. The testis stem cell niche consists of a cluster of quiescent somatic cells called the hub, which is anchored to the apical tip of the testis and supports two stem cell populations (Figure 1B). Germline stem cells (GSCs) divide to give rise to

© 2022 The Author(s).
This is an open access article under the CC BY license (http://creativecommons.org/licenses/by/4.0/).

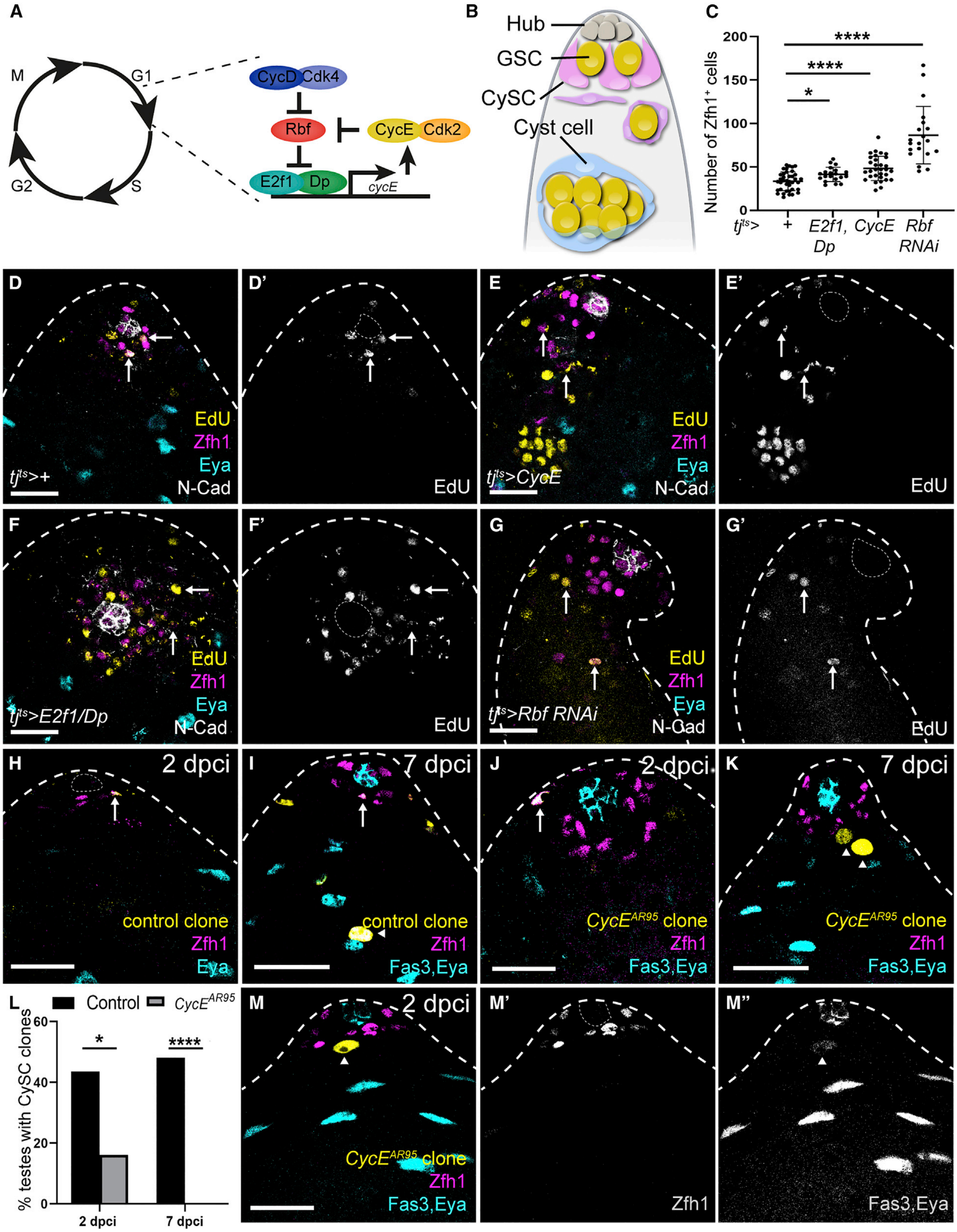

(legend on next page)

gonialblasts, which undergo a series of incomplete divisions to form a 16-cell cyst which matures and undergoes meiosis to form spermatids. A population of somatic stem cells, called cyst stem cells or CySCs, give rise to cyst cells, which ensheath the developing germline and support its differentiation (Hardy et al., 1979; Kiger et al., 2000; Tran et al., 2000). CySCs are the only proliferating somatic cells; their daughters exit the cell cycle as they differentiate (Cheng et al., 2011; Gonczy and DiNardo, 1996; Hardy et al., 1979), providing an ideal system to study how cell identity is linked to proliferative capacity. Previous work has shown a link between cell-cycle progression and CySC identity: CySCs lacking the Cdk activator Cdc25 (encoded by *string* in *Drosophila*) are not maintained (Inaba et al., 2011), while, conversely, accelerating proliferation results in an increased likelihood of self-renewal at the expense of neighboring wild-type CySCs (Albert et al., 2018; Amoyel et al., 2014, 2016; Michel et al., 2012). Moreover, mutants for the Rb homolog *Rbf* accumulate stem-like cells and lack differentiated cells in larval testes (Dominado et al., 2016). In adults, *Rbf* is required to maintain quiescence of terminally differentiated hub and cyst cells (Greenspan and Matunis, 2018), although its role in adult stem cells has not been established.

Since differentiating daughters of CySCs exit the cell cycle, we asked whether regulators of G1-S transition were important in maintaining CySC identity. We took advantage of reduced genetic redundancy in *Drosophila*, which has one activator E2f, called E2f1, and one repressor E2f, E2f2, both of which bind a single DP homolog, Dp, which mediates transcription by both activator and repressor complexes (Dynlacht et al., 1994; Frolov et al., 2001, 2005; Korenjak et al., 2012; Sawado et al., 1998). We find that E2f1/Dp activity in CySCs coordinates cell-cycle progression with stem cell identity by controlling CySC metabolism.

RESULTS

Promoting the G1/S transition causes ectopic proliferation and expands the CySC population

To test whether entry into S phase was linked to maintenance of CySC identity, we asked whether promoting progress through the G1/S transition could also affect cell fate. We manipulated the key cyclin controlling S phase entry, CycE, and the transcriptional regulator of S phase genes, the E2f activator complex, composed of E2f1 and Dp and their inhibitor, Rbf. We assessed cell proliferation by EdU incorporation and cell identity using antibodies against Zfh1, which labels CySCs and their immediate daughters (Leatherman and Dinardo, 2008), and Eya, which labels post-mitotic, differentiated cyst cells (Fabrizio et al., 2003). In control testes, Zfh1 expression was detected in 33.5 $\pm$ 1.7 cells (N = 38 testes) arranged in two tiers surrounding the hub, whereas cyst cells distant from the hub expressed Eya (Figures 1C and 1D). Consistent with previous reports that the only proliferating somatic cells in the testis are CySCs (Cheng et al., 2011; Gonczy and DiNardo, 1996; Hardy et al., 1979), DNA replication in somatic cells, as assayed by EdU incorporation, was only detected in Zfh1-positive cells around the hub (Figure 1D, arrows).

Using an endogenously tagged CycE-GFP fusion (Doherty et al., 2021), we detected CycE expression in occasional CySCs (Figure S1A, arrows), consistent with periodic expression during cell-cycle progression. We mis-expressed CycE in the cyst lineage using *traffic jam* (*tj*)*-Gal4*, which drives expression in CySCs and early cyst cells (Fairchild et al., 2016; Li et al., 2003), together with Gal80ts to restrict expression to adult stages (referred to as tj^{ts}). Mis-expressing CycE resulted in somatic cells distant from the hub incorporating EdU (Figure 1E, arrows), consistent with a role for CycE in promoting S phase entry. In addition, we observed an expansion of Zfh1-expressing cells away from the niche compared with controls (Figure 1E). The number of Zfh1-positive cells increased significantly in CycE-expressing testes (Kruskal-Wallis followed by Dunn's multiple comparison test, $p < 0.0001$, Figure 1C), from 33.5 $\pm$ 1.7 in controls (N = 38 testes) to 48.3 $\pm$ 2.5 (N = 31). However, we always observed Eya-positive cells further distally (Figure 1E), and never observed EdU incorporation in these cyst cells, suggesting that, although delayed, differentiation occurred normally in somatic cells overexpressing CycE.

Next, we tested whether the transcriptional regulator of S phase gene expression, E2f1, together with its partner Dp, could influence CySC fate. Previous work has shown that E2f1/Dp is active in CySCs (Amoyel et al., 2014; Herrera et al., 2021). Using an established reporter for E2f1/Dp transcriptional activity, *PCNA-GFP* (Thacker et al., 2003), we detected E2f1/Dp activity in CySCs (Figure S1B, arrows), but not in differentiated cyst cells away from the hub (Figure S1B, arrowheads, quantified in Figure S1C). In CySCs, *PCNA-GFP* was detected in a subset of cells, consistent with periodic cell-cycle-dependent activation

Figure 1. Regulators of the G1/S transition affect CySC numbers and self-renewal
(A) Diagram of regulatory interactions controlling S phase entry.
(B) Schematic of the *Drosophila* testis. The hub (gray) supports germline stem cells (GSCs) (yellow) and somatic cyst stem cells (CySCs) (magenta). CySCs produce post-mitotic cyst cells (cyan) which support germ cell development.
(C) Quantification of the number of Zfh1$^+$, Eya$^-$ cells in the genotypes indicated.
(D–G) Testes from control (D) or from animals mis-expressing CycE (E), E2f1/Dp (F), or Rbf RNAi (G) in somatic cells with tj^{ts}>, labeled with antibodies against Zfh1 (magenta) to mark CySCs and early daughter cells, N-Cad (white) to label the hub, Eya (cyan) to label differentiated cyst cells, and EdU (yellow) (D').
(H–K) Control (H and I) or *CycE* (J and K) mutant clones positively labeled with GFP (yellow) and identified as CySCs by Zfh1 expression (magenta) or cyst cells using Eya (cyan).
(H) Control clones were recovered at 2 days post clone induction (dpci) and maintained at 7 dpci (I).
(J) $CycE^{AR95}$ mutant CySCs at 2 dpci (arrow).
(K) $CycE^{AR95}$ mutant clones at 7 dpci with only Eya$^+$ cells (arrowheads). Note the enlarged nucleus of mutant cells.
(L) Fraction of testes containing marked control or $CycE^{AR95}$ mutant CySC clones. See Table S1 for N values.
(M) $CycE^{AR95}$ mutant cells at 2 dpci expressed Eya (M'') prematurely and downregulated Zfh1 (M'). *$p < 0.05$, ****$p < 0.0001$, determined by Kruskal-Wallis and Dunn's multiple comparisons (C) or Fisher's exact test (L). Dotted lines outline the hub. Scale bars, 20 μm.

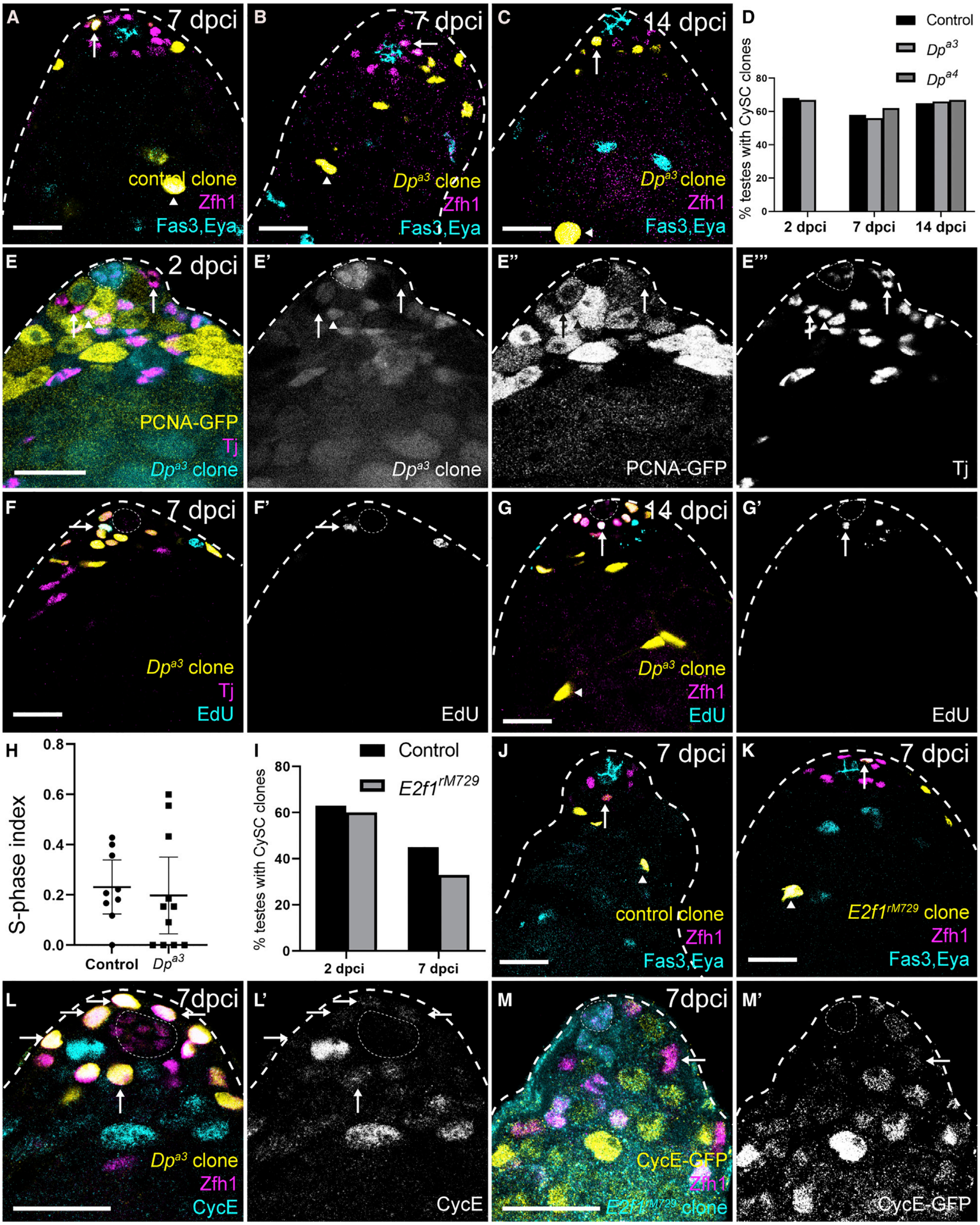

(legend on next page)

of E2f1/Dp. Reporter expression was strongly reduced upon Dp knockdown (Figures S1D–S1F), indicating that *PCNA-GFP* expression reflects endogenous Dp-dependent transcription. Similar to CycE overexpression, Dp and E2f1 overexpression led to ectopic proliferation of somatic cells far from the hub (Figure 1F, arrows). In addition, we counted 41.4 ± 1.8 Zfh1-positive cells, significantly higher than the control (N = 20, Kruskal-Wallis followed by Dunn's multiple comparison test, $p < 0.046$, Figure 1C). To test whether activating endogenous E2f1/Dp could also result in ectopic Zfh1-expressing cells, we knocked down the negative regulator of this complex, Rbf. Rbf expression was detected in all cells at the apical tip of the testes (Figure S1G), as described previously (Greenspan and Matunis, 2018), and efficient knockdown was achieved by RNAi expression (Figure S1H). Expression of an RNAi against *Rbf* in the somatic lineage led to an expansion of the Zfh1-positive population to 86.6 ± 7.4 (N = 20, Kruskal-Wallis followed by Dunn's multiple comparison test, $p < 0.0001$), in addition to ectopic proliferation away from the hub (Figures 1C and 1G). Importantly, testes in which Rbf was knocked down had no Zfh1-negative, Eya-positive cyst cells (Figure 1G and see below), implying a complete block in differentiation.

Altogether, over-activating key drivers of the G1/S transition is sufficient to promote proliferation away from the stem cell niche and affects the ability of CySCs to differentiate.

CycE is required for CySC self-renewal

Next, we asked whether these regulators were necessary for the maintenance of CySC identity. We generated marked CySC clones using mitotic recombination. Since CySCs are the only dividing somatic cells in the testis, any labeled somatic cells were necessarily generated from a CySC division. We measured the persistence of clones over time as a reflection of the ability of labeled CySCs to self-renew in the niche. Control marked clones were readily recovered at 2 days post clone induction (dpci) (Figures 1H and 1L; Table S1) and were maintained at 7 dpci (Figures 1I and 1L; Table S1).

By contrast, clones mutant for a null allele of *CycE* never contained Zfh1-expressing CySCs at 7 dpci and all clones consisted exclusively of Eya-positive cyst cells (Figures 1J–1L; Table S1). The impaired self-renewal of *CycE* mutant CySCs was already evident at 2 dpci, as few CySC clones were observed at that stage ($p < 0.035$, Fisher's exact test, Figures 1J and 1L; Table S1). By 7 dpci, only Eya-positive differentiated cells were labeled ($p < 0.0001$, Fisher's exact test, Figure 1K, arrowheads). We note that Eya-positive *CycE* mutant cells did not appear wild-type, as their nuclei were enlarged, similar to reports in the female germline (Ables and Drummond-Barbosa, 2013). Despite low CySC clone recovery rates, even at 2 dpci, clones were induced at similar rates to controls, as GFP-positive *CycE* mutant somatic cells were observed in 26/31 (or 84%) testes examined compared with 20/23 (87%) in controls ($p > 0.99$, Fisher's exact test). However, most of these cells did not express Zfh1, suggesting that *CycE* mutant CySCs differentiated rapidly. To confirm our findings, we generated clones homozygous mutant for a hypomorphic allele. $CycE^{WX}$ homozygous CySC clones were recovered significantly less than control clones at 7 dpci (Table S1, $p < 0.041$, Fisher's exact test), consistent with a requirement for *CycE* in CySC self-renewal. We used an antibody against the activated effector caspase, Death caspase-1, but did not observe any dying clonal CySCs either in control (N = 21 clones) or in *CycE* mutant clones (N = 9 clones). In contrast, by 2 dpci, we observed mutant clones that expressed Eya prematurely (Figure 1M, arrowhead), surrounded by wild-type cells expressing Zfh1. These observations suggest that *CycE* mutant CySC clones are poorly recovered because they differentiate prematurely.

Taken together with the gain-of-function experiments described above, our data indicate that *CycE* is necessary for CySC self-renewal and at least partly sufficient to drive ectopic Zfh1 expression and cell-cycle progression several cell diameters away from the hub, establishing *CycE* as a critical regulator of CySC fate.

The E2f1/Dp complex is not required for CySC self-renewal

In most contexts studied to date, *CycE* expression is transcriptionally induced at the G1/S transition by the E2f1/Dp complex (Dimova and Dyson, 2005; Dimova et al., 2003; Duronio et al., 1995, 1996; Korenjak et al., 2012; Stevaux and Dyson, 2002; Vermeulen et al., 2003). Since *CycE* is essential for CySC maintenance, we reasoned that *E2f1* and *Dp* would also be required for CySC self-renewal and generated mutant clones to assess their role.

Unexpectedly, both control and *Dp* mutant CySC clones were recovered at 7 and 14 dpci (Figures 2A–2C) with indistinguishable

Figure 2. Dp and E2f1 are dispensable for CySC self-renewal

(A–C, F, G, K, and J) Positively marked control (A and J), *Dp* mutant (B, C, F, and G), or *E2f1* mutant (K) CySCs labeled with GFP expression (yellow) at the indicated dpci. CySCs were identified by Zfh1 expression (magenta) and position adjacent to the hub (Fas3, cyan). Differentiated cyst cells were labeled with Eya (cyan).

(D) Fraction of testes containing marked control or *Dp* mutant CySC clones. Clone recovery rates were not significantly different at 7 and 14 dpci, determined by Fisher's exact test. See Table S1 for N values.

(E) Negatively marked *Dp* mutant clones (arrow) at 2 dpci labeled by lack of RFP expression (cyan) (E'), showing decreased levels of PCNA-GFP expression (yellow) (E'') compared with wild-type CySCs (arrowhead). Somatic cells are labeled with Tj (magenta) (E''').

(F and G) Positively labeled *Dp* mutant clones at 7 dpci (F) and 14 dpci (G) incorporated EdU (cyan) (F' and G').

(H) S phase index of control and *Dp* mutant clones. No differences were observed using a Mann-Whitney test.

(I–K) Control (J) and *E2f1* mutant clones (K) at 7 dpci.

(I) Fraction of testes containing control or $E2f1^{rM729}$ CySC clones. Mutant clone recovery rates were not different from controls, as determined by Fisher's exact test.

(L) CycE (cyan) (L') detected in *Dp* mutant CySCs, positively marked by GFP expression (yellow, arrows).

(M) CycE-GFP expression (yellow) (M') in *E2f1* mutant clones, marked by the loss of RFP (cyan, arrow). See Table S1 for N values. Dotted lines outline the hub. Scale bars, 20 μm.

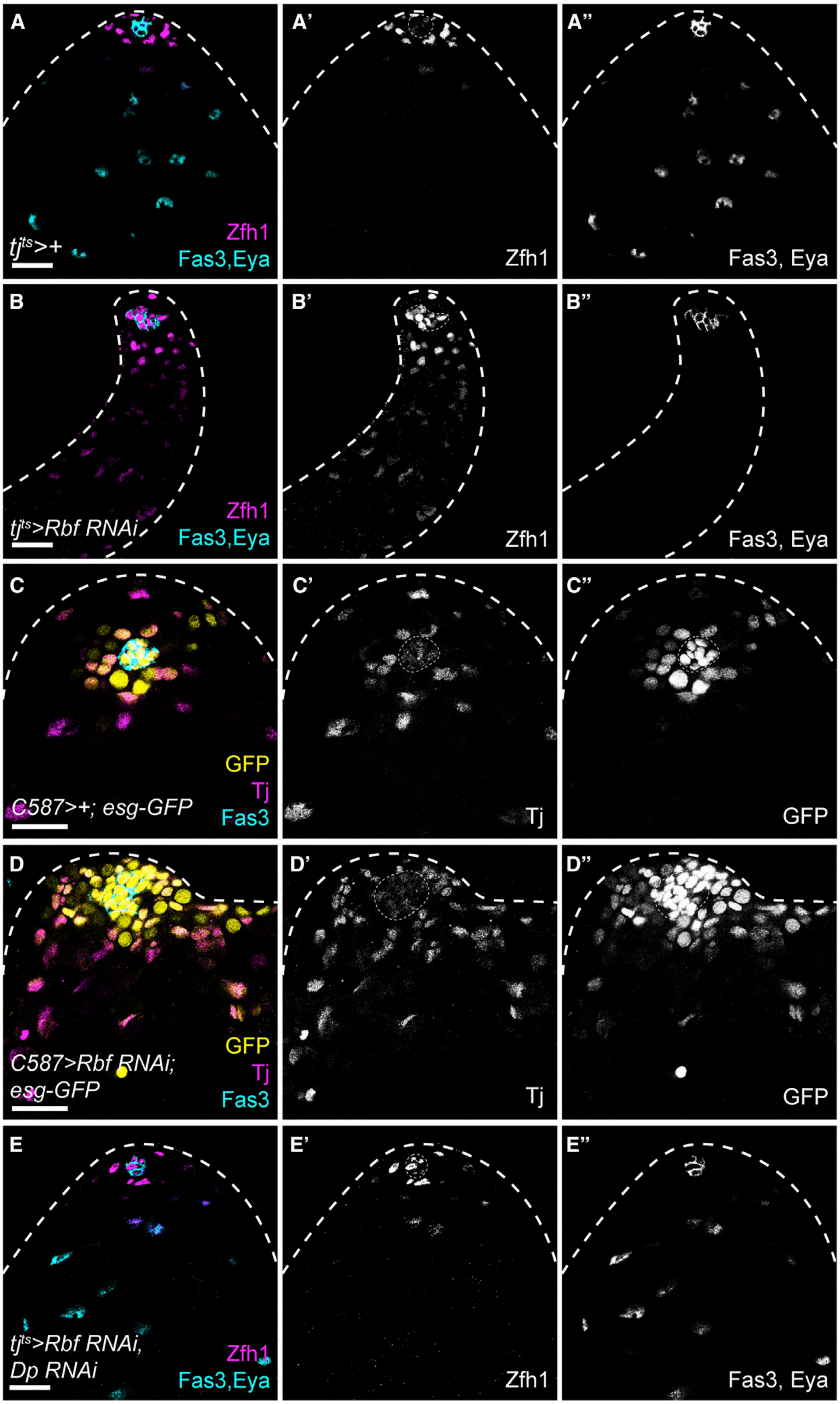

(legend on next page)

CellPress
OPEN ACCESS

clone recovery rates (p > 0.99 at both 7 and 14 dpci for *Dp*^{a3} and p = 0.72 at 7 dpci and p > 0.99 at 14 dpci for *Dp*^{a4} compared with control, Fisher's exact test, Figure 2D). We confirmed this surprising result with two separate null alleles (Figure 2D; Table S1), and verified genetically that the alleles we used were indeed *Dp* mutants and did not complement a deficiency uncovering the *Dp* locus (see Supplemental materials and methods). Moreover, no Dp protein could be detected in *Dp* mutant clones (Figure S1I). In addition, we examined the expression of the Ef21/Dp transcriptional target *PCNA* in *Dp* mutant clones and observed reduced reporter expression (Figure 2E). *Dp* mutant clones at 7 and 14 dpci contained many cells and could incorporate EdU (Figures 2F and 2G), indicating that *Dp* is dispensable for DNA replication and proliferation in CySCs. Indeed, the S phase index of *Dp* mutant clones was not different to that of control clones at 7 dpci (Figure 2H). Although surprising, this result is consistent with work showing that most tissues in *Drosophila* can proliferate in the absence of Dp activity during development (Frolov et al., 2001; Royzman et al., 1997; Zappia and Frolov, 2016).

Consistently, *E2f1* mutant CySC clones were recovered at 7dpci (Figures 2I–2K, arrows) with a slightly lower but not significantly different clone recovery rate to controls (Fisher's exact test, p > 0.05, Figure 2I). Like control and *Dp* mutant clones, *E2f1* mutant clones were composed of both Zfh1-expressing CySCs and Eya-positive differentiating cyst cells (Figures 2J and 2K, arrows and arrowheads, respectively), suggesting that neither their self-renewal nor their differentiation capacity was altered.

Finally, we tested whether Cdk4, an upstream positive regulator of E2f1/Dp, was required for CySC self-renewal. We induced mutant clones for two separate alleles of *Cdk4*. These clones were recovered at similar rates to controls at 7 dpci (Figure S2; Table S1), indicating that *Cdk4* is not required for CySC self-renewal.

Altogether, our data show that E2f1/Dp activity is not required in CySCs for self-renewal, despite the fact that ectopically activating this complex is sufficient to drive CySC over-proliferation (Figure 1). This result stands in contrast with the requirement identified above for *CycE* in CySC self-renewal and implies that *CycE* expression in CySCs does not depend on E2f1/Dp or CycD/Cdk4 activity. Indeed, we observed that both *Dp* and *E2f1* mutant CySCs could express CycE, as detected by an antibody or endogenously tagged CycE-GFP (Figures 2L and 2M).

E2f1/Dp inhibition by Rbf is required for cyst cell differentiation

Despite a genetic lack of requirement for *Dp* in CySC self-renewal, Dp-dependent transcriptional activity was detected in CySCs (Figure S1) (Amoyel et al., 2014) in a pattern suggesting cell-cycle-dependent E2f1/Dp activation. Moreover, Rbf knockdown resulted in a block of CySC differentiation (Figure 1F), suggesting that E2f1 and Dp were indeed expressed in the cyst lineage. Although E2f1/Dp overexpression resulted in a weaker phenotype than Rbf knockdown (Figures 1C, 1F, and 1G), this could be due to the presence of endogenous Rbf or to insufficient expression levels. To resolve the question of what endogenous role Rbf and E2f1/Dp activity might play in CySC self-renewal and differentiation, we characterized the role of *Rbf* in CySCs and examined the dependency of Rbf knockdown on E2f1/Dp.

Lineage-wide Rbf knockdown with *tj*^{ts} led to expansion of Zfh1-expressing cells such that they filled the entire testis, and an absence of Eya-positive differentiated cyst cells (Figures 1F, 3A, and 3B). Similarly, CySC clones overexpressing two different RNAi constructs targeting Rbf were entirely composed of Zfh1-expressing cells and devoid of Eya-expressing cells (Figures S3A–S3D). Finally, we generated negatively marked CySC clones hemizygous mutant for the loss-of-function allele *Rbf*^{14} (see STAR Methods). CySC clones mutant for *Rbf* displayed a similar phenotype to Rbf knockdown CySCs: clones were only composed of Zfh1-expressing cells and no Eya-expressing differentiated cyst cells were present within the clones (Figures S3E and S3F, N = 22/26), indicating a block in differentiation in *Rbf* mutant CySCs.

The ectopic Zfh1-expressing, Eya-negative cells that were detected away from the niche in Rbf knockdowns expressed low levels of Zfh1 compared with cells adjacent to the hub (Figures 1F and 3B). To confirm whether these ectopic cells were indeed CySCs, we examined other markers of CySCs, Esg, Chinmo, and Wg (Flaherty et al., 2010; Leatherman and Dinardo, 2008; Voog et al., 2014). In controls, both Esg and Chinmo labeled CySCs, as well as the hub and early germ cells (Figures 3C and S4A). Both markers were expanded throughout Rbf knockdown testes and showed an expression pattern similar to Zfh1 (Figures 3D and S4B), with high expression in CySCs close to the hub and lower levels in ectopic cells throughout the testis. Wg expression was observed in distinctive puncta around the hub in control testes, but punctate expression was observed throughout the testis in Rbf knockdowns (Figures S4C and S4D). These results suggest that Rbf-depleted cells maintain a CySC-like state similar to immediate CySC daughters, which have lower Zfh1 expression than CySCs in contact with the hub (Leatherman and Dinardo, 2008), but that their differentiation does not progress further. Furthermore, preventing cell death could not restore differentiation in the Rbf knockdowns and activation of the self-renewal pathway JAK/STAT was not responsible for the presence of ectopic CySCs upon Rbf loss (Figures S4E–S4J).

Previous work has shown that, in larvae, Rbf binding to DNA is abolished in the absence of Dp (Korenjak et al., 2012), indicating that Rbf exerts its effects on gene expression through Dp.

Figure 3. Inhibition of E2f1/Dp is necessary for cyst cell differentiation

(A and B) Control testis (A) or Rbf knockdown (B) showing Zfh1 expression (magenta) (A') in CySCs surrounding the hub labeled with Fas3 and Eya (cyan) (A'') in cyst cells.

(C and D) *esg-GFP* expression (yellow) (C'' and D'') in control (C) and Rbf knockdown (D) testes. The hub is labeled with Fas3 (cyan), and CySCs and early cyst cells are labeled with Tj (magenta) (C' and D').

(E) Knockdown of both Dp and Rbf resulted in Zfh1 expression (magenta) (E') being restricted around the hub (Fas3, cyan) and Eya^{+} cells detected further from the hub (cyan) (E'') similar to control testes. Dotted lines outline the hub. Scale bars, 20 μm.

Indeed, loss of function of both *Dp* and *Rbf* led to a similar distribution of Zfh1-expressing cells around the hub and Eya-expressing cells away from the hub as in control testes, both in tissue-wide knockdowns (Figure 3E) and in mutant clones (Figures S3D and S3H), despite the lack of any detectable Rbf protein by immunohistochemistry (Figure S3I, arrowhead). Consistently, *E2f1* loss of function also restored differentiation in Rbf RNAi clones (Figures S3D and S3G), similar to observations in larval testes (Dominado et al., 2016). Thus, the effects of *Rbf* loss of function in CySCs are attributable entirely to ectopic E2f1/Dp activity.

In sum, our data suggest that the E2f1/Dp complex acts as a link to coordinate cell cycle and CySC identity. While E2f1/Dp activity is not required for cell-cycle progression, its continued activity in CySCs is sufficient to inhibit differentiation. In turn, Rbf acts as a permissive factor for differentiation by relieving the E2f1/Dp-mediated inhibition of differentiation.

Ectopic E2f1/Dp activity in CySCs alters expression of genes regulating metabolism and energy production

To gain insight into the mechanisms by which the E2f1/Dp complex transcriptionally inhibits differentiation, we compared gene expression in sorted somatic cells in *tj*>+ control and Rbf knockdown testes. Using stringent criteria (2-fold change and FDR < 0.01), we identified >5,000 differentially expressed transcripts (3,329 upregulated and 2,098 downregulated in Rbf knockdown compared with control) (Table S2), indicating that Rbf knockdown results in a large disruption to gene expression, presumably as a combination of the direct effects of Rbf loss and indirect effects of a block in differentiation.

Gene ontology (GO) analysis of upregulated genes revealed an enrichment for several processes involved in cell-cycle progression, DNA synthesis, and replication (Figure 4A). The latter category included many well-described E2f1/Dp targets, such as *PCNA* and *Minichromosome maintenance* (Mcm) genes (Ishida et al., 2001; Yamaguchi et al., 1995) (Figure 4B). In particular, *PCNA* was upregulated 20-fold following Rbf knockdown (Table S2), which we confirmed using the *PCNA-GFP* reporter (Figures 4C–4E). In controls, GFP in the somatic lineage was observed only adjacent to the hub (Figure 4C, arrow), whereas it was absent far from the hub (Figure 4C, arrowhead). However, in Rbf knockdowns, GFP was detected in somatic cells distant from the hub (Figure 4D, arrows). In addition to cell-cycle-related genes, expression of CySC and early cyst cell markers was also differentially detected in the Rbf knockdown, including *Zfh1* (10.4-fold increase, FDR < 0.001), *chinmo* (5.2-fold increase, FDR < 0.001), and *tj* (17.9-fold increase, FDR < 0.001). These results recapitulate our previous observations that these markers were ectopically expressed in Rbf knockdowns (Figures 1G, 3B, and S4B).

While upregulated transcripts were largely consistent with increased proliferation, transcripts that were downregulated upon Rbf knockdown fell into distinct categories (Figure 5A). Transcripts encoding cell junction proteins were downregulated, in keeping with previous reports showing that expression of septate and adherens junction components increases during cyst cell differentiation (Dubey et al., 2019; Fairchild et al., 2015, 2017; Papagiannouli et al., 2019). A large number of downregulated transcripts encoded genes involved in various aspects of oxidative metabolism and ATP production (Figures 5A and 5B), suggestive of an altered metabolic state in Rbf-deficient cells. To validate the downregulation observed by sequencing, we examined the expression of a GFP protein trap in the *Aldolase 1* (*Ald1*) locus, which was downregulated in the Rbf knockdown group. In controls, Ald1-GFP expression was detected at low levels in CySCs and increased distally from the hub (Figure 5C). Expression was reduced in both CySCs adjacent to the hub and ectopic CySC-like cells in testes in which Rbf was knocked down somatically (Figure 5D). Since the reduction in Ald1-GFP away from the hub might reflect a lack of differentiated cells, we quantified GFP fluorescence in control and Rbf-deficient CySCs adjacent to the hub. We observed a significant decrease in Ald1-GFP in CySCs in which Rbf was knocked down (Figure 5E, $p < 0.0001$, Mann-Whitney test).

The reduction in Ald1 expression in Rbf-deficient CySCs suggested that they may have a metabolic defect prior to differentiation. Therefore, we examined mitochondrial morphology in control and Rbf-deficient CySCs using electron microscopy. In control testes, the cytoplasm of CySCs contained numerous mitochondria (Figure 5F). Mitochondria in differentiated cyst cells were more elongated and more electron dense than in CySCs (Figure 5G). By contrast, in Rbf knockdown CySCs we observed many fewer mitochondria, and these appeared smaller and more globular than in controls (Figure 5H). Somatic cells located away from the hub displayed similar small and round mitochondria and had many fewer mitochondria present than control differentiated cyst cells, resembling more closely the distribution and morphology observed in Rbf-deficient CySCs (Figure 5I). To confirm these observations, we used the dye tetramethylrhodamine (TMRM), to assess mitochondrial membrane potential (Figures 5J–5L). We quantified TMRM intensity in CySCs contacting the hub in control and Rbf knockdown testes, and observed a significant reduction in Rbf knockdowns (Figure 5L, $p < 0.0001$, Mann-Whitney test).

Overall, our data show that Rbf-deficient CySCs express lower levels of genes controlling many aspects of oxidative metabolism than control CySCs, and have reduced mitochondrial activity. These results suggest that lower metabolic activity in Rbf-deficient cells may contribute to their inability to differentiate and raise the possibility that alterations in metabolism may play a role in normal cyst cell differentiation.

Endogenous cyst cell differentiation is associated with metabolic gene expression changes

To test whether wild-type cyst cell differentiation did indeed involve increased expression of metabolic genes, we compared gene expression profiles in sorted CySCs (Figure S5A) and differentiating cyst cells (Figure S5B) in control animals. Principal-component analysis considering all expressed genes separated the ten transcriptomes into two non-overlapping clusters (Figure S5C). Applying stringent criteria of FDR $< 10^{-3}$ and an absolute of the $\log_2$(fold change) > 1.5, we found 571 upregulated genes in CySCs compared with the cyst cell population and 1,284 downregulated ones (Table S2). We examined known CySC and cyst cell markers to validate our results. *zfh1* transcripts were detected at a 5.4-fold higher level in the CySC

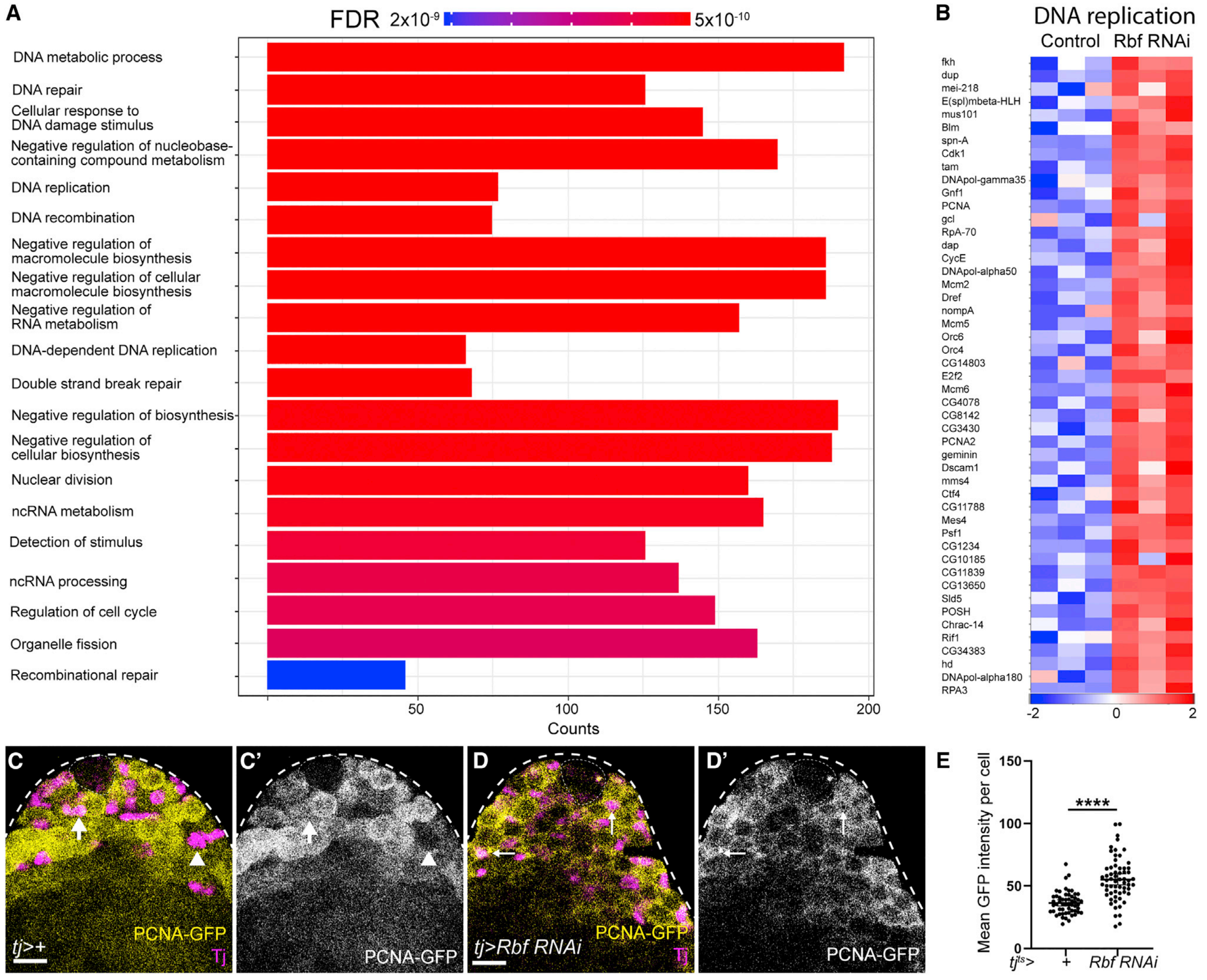

Figure 4. Rbf knockdown results in upregulation of genes involved in cell-cycle progression and DNA replication

(A) Plot showing the most significantly enriched gene ontology (GO) terms among genes upregulated upon Rbf knockdown. Significance is indicated by color coding from red to blue, and the length of each column reflects the number of genes.

(B) Heatmap showing relative expression of genes grouped under the GO term "DNA replication" for control and Rbf knockdown testes. Columns represent biological replicates. Blue represents lower expression in $\log_2$(fold change), while higher expression is shown in red.

(C and D) Expression of the *PCNA-GFP* reporter (yellow) (C' and D') in control (C) and Rbf knockdown (D) testes. Tj (magenta) labels CySCs and early cyst cells.

(E) Quantification of mean GFP intensity in CySCs reveals a significant increase in PCNA-GFP intensity in Rbf knockdown. ****$p < 0.0001$, determined by Mann-Whitney test. Dotted lines outline the hub. Scale bars, 20 μm.

samples relative to differentiated cyst cells (Figure S5D, and see Table S2). Consistent with previous observations (Inaba et al., 2011; Li et al., 2003), Zfh1-positive CySCs exhibited 2.2-fold higher expression of *tj* and 1.6-fold expression of *string* (*stg*) than Zfh1-negative differentiated cyst cells (Figures S5D and S5E; Table S2). Conversely, expression of the differentiation marker *eya* (Fabrizio et al., 2003) was upregulated 6.2-fold in differentiated cyst cells relative to CySCs, while *Rbf* expression was 12.5-fold higher (Figures S5D and S5E).

Having validated that our approach enabled us to identify genes that were differentially expressed in and relevant to the function of CySCs, we next asked what transcriptional changes occurred during normal cyst cell differentiation. Genes upregulated in cyst cells relative to CySCs were enriched for GO terms associated with all aspects of cellular energy generation through oxidative phosphorylation, including pyruvate metabolism, TCA cycle, and the mitochondrial electron transport chain (Figures 6A and 6B). These data are consistent with the more mature mitochondrial morphology visible in differentiated cyst cells by electron microscopy (Figures 5F and 5G). These differences in gene expression suggested that cyst cells and CySCs differed in their cell physiology, in particular that differentiated cyst cells had a metabolic state biased toward increased oxidative phosphorylation. To test this, we used the MitoTimer sensor (Laker et al.,

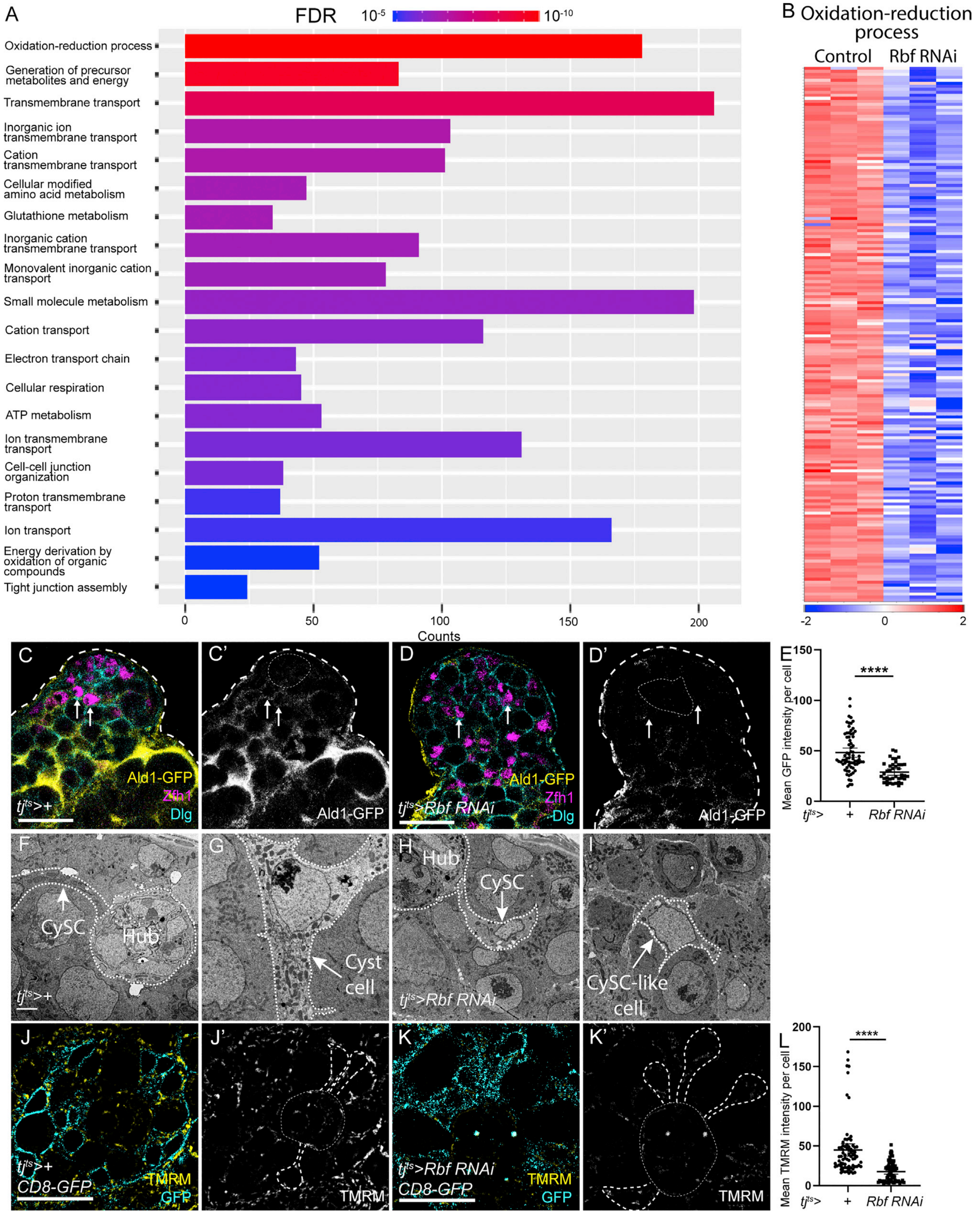

(legend on next page)

2014), which consists of a mitochondrially targeted RFP that is initially present in an immature green fluorescent precursor but transitions to the mature red fluorescent form when oxidized. Using *tj*^{ts} to express the reporter in a single overnight pulse, we observed that the mitochondrial matrix of differentiated cyst cells had a lower ratio of reduced to oxidized MitoTimer than CySCs (Figures 6C–6E, $p < 0.001$, t test), suggesting increased oxidation in the mitochondrial matrix as cyst cells differentiate. To validate this result, using a dye that was independent of driver expression, we examined the pattern of TMRM labeling in CySCs and differentiated cyst cells (Figures 6F and 6G). We measured the aspect ratio of mitochondria and observed a higher circularity index in CySCs compared with cyst cells (Figure 6H, $p < 0.0003$, Mann-Whitney test), consistent with cyst cells having more elongated and complex mitochondrial networks. TMRM intensity was significantly higher in cyst cells than CySCs (Figure 6I, $p < 0.0001$, Mann-Whitney test), indicating increased mitochondrial activity in differentiated cells.

Altogether, differentiation of wild-type CySCs into cyst cells involves significant transcriptional changes of genes regulating cellular metabolism, resulting in increased mitochondrial activity and higher morphological complexity with differentiation.

Promoting mitochondrial biogenesis can rescue differentiation but not cell-cycle exit in Rbf knockdown testes

Our results indicate that oxidative metabolism increases during cyst cell differentiation, and that, conversely, CySCs lacking Rbf have decreased expression of genes encoding mitochondrial proteins and fewer mitochondria. We asked how similar the gene expression profile of Rbf-deficient CySCs was to wild-type CySCs. Approximately 27% of genes that were differentially expressed between CySCs and cyst cells were similarly changed in Rbf-deficient CySCs (Figure S6A; Table S2). In addition, GO analysis of the overlapping downregulated genes revealed enrichment for categories related to energetic metabolism and oxidative phosphorylation (Figure S6B). Genes downregulated in these categories include glycolytic and mitochondrial enzymes, such as *Ldh*, *PyK*, *Gapdh1*, *blw*, or *Mtpα*. These data suggest that the gene expression of Rbf-deficient CySCs is similar to wild-type CySCs for metabolic genes and that the metabolic state of Rbf-deficient CySCs may be limiting their ability to differentiate.

Since Rbf-deficient CySCs showed decreased expression of metabolic genes and fewer mitochondria, we asked whether promoting mitochondrial biogenesis in Rbf-deficient CySCs could rescue their block in differentiation (Figures 7A and 7B). To this end, we expressed the transcription factors Spargel (Srl, homolog of PGC1α), which, together with Delg (Flybase: Ets97D, the homolog of NRF-2α), non-redundantly regulate mitochondrial gene expression and promote mitochondrial biogenesis and activity (Rera et al., 2011; Tiefenbock et al., 2010). In otherwise wild-type CySCs, overexpression of Srl and Delg led to an increase in staining for the mitochondrial dye MitoTracker, indicating higher mitochondrial mass (Figures 7C and S7A). However, we observed no difference in the intensity of TMRM fluorescence compared with control (Figure 7D), suggesting that Srl and Delg expression specifically increased mitochondrial mass but not activity in CySCs. We next assessed whether overexpression of Srl and Delg could increase mitochondrial mass or activity in Rbf-deficient CySCs. Indeed, we observed that both MitoTracker and TMRM intensity were significantly increased in Rbf-deficient CySCs upon expression of Srl and Delg (Figures 7E, 7F, and S7B). Thus, driving mitochondrial biogenesis in CySCs lacking Rbf is sufficient to partially restore mitochondrial activity levels.

Finally, we tested whether expression of Srl and Delg could rescue differentiation in Rbf-deficient cells. While knockdown of Rbf resulted in expansion of Zfh1 expression away from the hub and a lack of Eya-expressing cells (Figures 7A and 7B), co-expression of Srl and Delg led to a distribution of cell types that resembled control testes: Zfh1-expressing cells were restricted to about two rows surrounding the hub, while cells further away expressed Eya (Figure 7G). We counted Zfh1-expressing cells and found that, whereas knockdown of Rbf resulted in an increase from 33.5 ± 1.7 in controls to 86.6 ± 7.4 (Figure 7H, $p < 0.0001$, Kruskal-Wallis and Dunn's multiple comparisons test, $N = 38$ for controls and $N = 20$ for Rbf knockdown), this was significantly reduced to 41.1 ± 2.1 Zfh1-positive cells when Srl and Delg were co-expressed ($p < 0.0001$ compared with Rbf knockdown, $p < 0.1$ compared with control, Dunn's multiple comparisons, $N = 37$). In addition, while in Rbf knockdowns only 10% of testes contained Eya-positive differentiated cyst cells ($p < 0.0001$ compared with control, Fisher's exact test), all rescued testes contained Eya-positive cells (Figure 7I, $p < 0.0001$ compared with Rbf knockdown, Fisher's exact

Figure 5. Rbf knockdown downregulates genes related to oxidative metabolism and energy production

(A) Plot showing the most significantly enriched GO terms among genes downregulated upon Rbf knockdown. Significance is indicated by color coding from red to blue, and the length of each column reflects the number of genes.

(B) Heatmap showing relative expression of genes grouped under the GO term "oxidation-reduction process" for control and Rbf knockdown testes. Columns represent biological replicates. Blue represents lower expression in $\log_2$(fold change), while higher expression is shown in red.

(C and D) Expression of a GFP protein trap for the glycolytic enzyme Aldolase1 (Ald1-GFP, yellow) (C' and D') in control (C) and Rbf knockdown (D) testes. Zfh1 (magenta) labels CySCs and Dlg (cyan) labels cell outlines.

(E) Quantification of Ald1-GFP intensity in CySCs immediately adjacent to the hub. ****$p < 0.0001$, as determined by Mann-Whitney test.

(F–I) Electron micrographs of the testis apex in controls (F and G) and Rbf RNAi (H and I). Many mitochondria are visible in a control CySC (F) (arrow). In control differentiating cyst cells (G), numerous electron-dense, elongated mitochondria can be seen. In Rbf-deficient CySCs (H) and CySC-like cells distant from the hub (I), few mitochondria are visible.

(J and K) Testes from *tj*^{ts} > *CD8-GFP* animals stained with tetramethylrhodamine (TMRM) (yellow) (J' and K') to label active mitochondria in control (J) and Rbf knockdowns (K). CySCs (dashed lines) adjacent to the hub (dotted line) were outlined using GFP expression (yellow).

(L) TMRM intensity was significantly reduced in Rbf-deficient CySCs compared with controls. ****$p < 0.0001$ as determined by Mann-Whitney test. Scale bars, 20 μm in (C, D, J, and K), and 2 μm in (E–H).

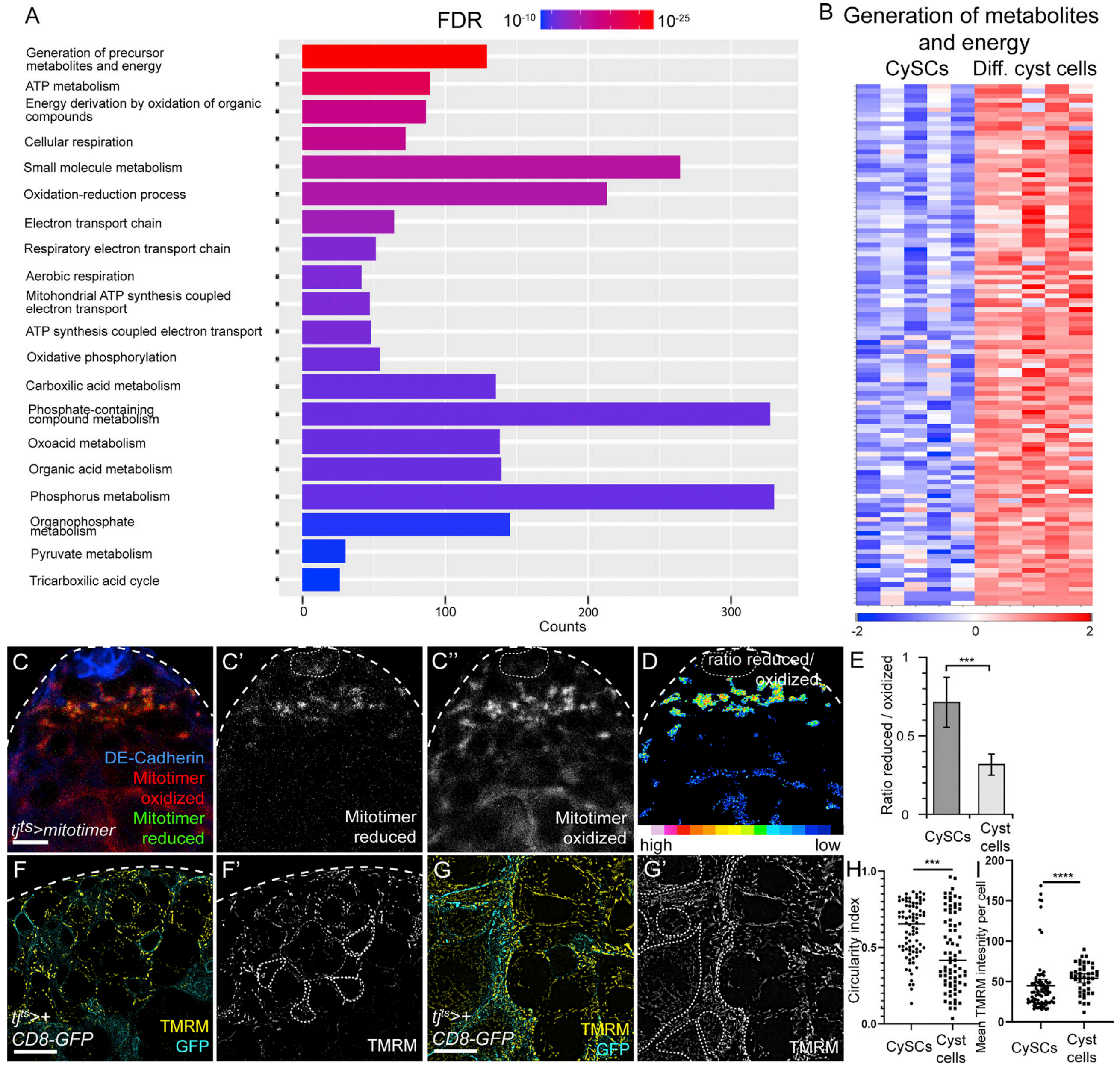

Figure 6. Endogenous cyst cell differentiation involves changes in metabolic gene expression and activity

(A) Plot showing the most significantly enriched GO terms among genes downregulated in control CySCs compared with differentiating cyst cells. Significance is indicated by color coding from red to blue, and the length of each column reflects the number of genes contained within each GO term.

(B) Heatmap showing relative expression of genes grouped under the GO term "generation of metabolites and energy" in CySCs and differentiating cyst cells. Columns represent biological replicates. Blue represents lower expression in $\log_2$(fold change), while higher expression is shown in red.

(C) Expression of MitoTimer in a control testis driven with tj^{ts}. Oxidized (red) (C'') reporter is detected both in CySCs close to the hub (DE-Cadherin, blue) and in cyst cells distant from it. Reduced (green) (C') reporter was mainly present in CySCs surrounding the niche.

(D) Ratio of reduced/oxidized MitoTimer.

(E) Quantification of the ratio of reduced to oxidized MitoTimer. ***$p < 0.001$, N = 19 testes, Student's t test.

(F and G) Testes from tj^{ts} > *CD8-GFP* animals stained with TMRM (yellow) (F' and G') in control CySCs (F) and differentiated cyst cells (G). GFP (cyan) was used to outline individual CySCs and cyst cells.

(H) Circularity index of mitochondria in control CySCs and cyst cells.

(I) Quantification of TMRM intensity in control CySCs and cyst cells. ***$p < 0.001$, ****$p < 0.0001$, determined by Mann-Whitney test. Scale bars, 20 μm.

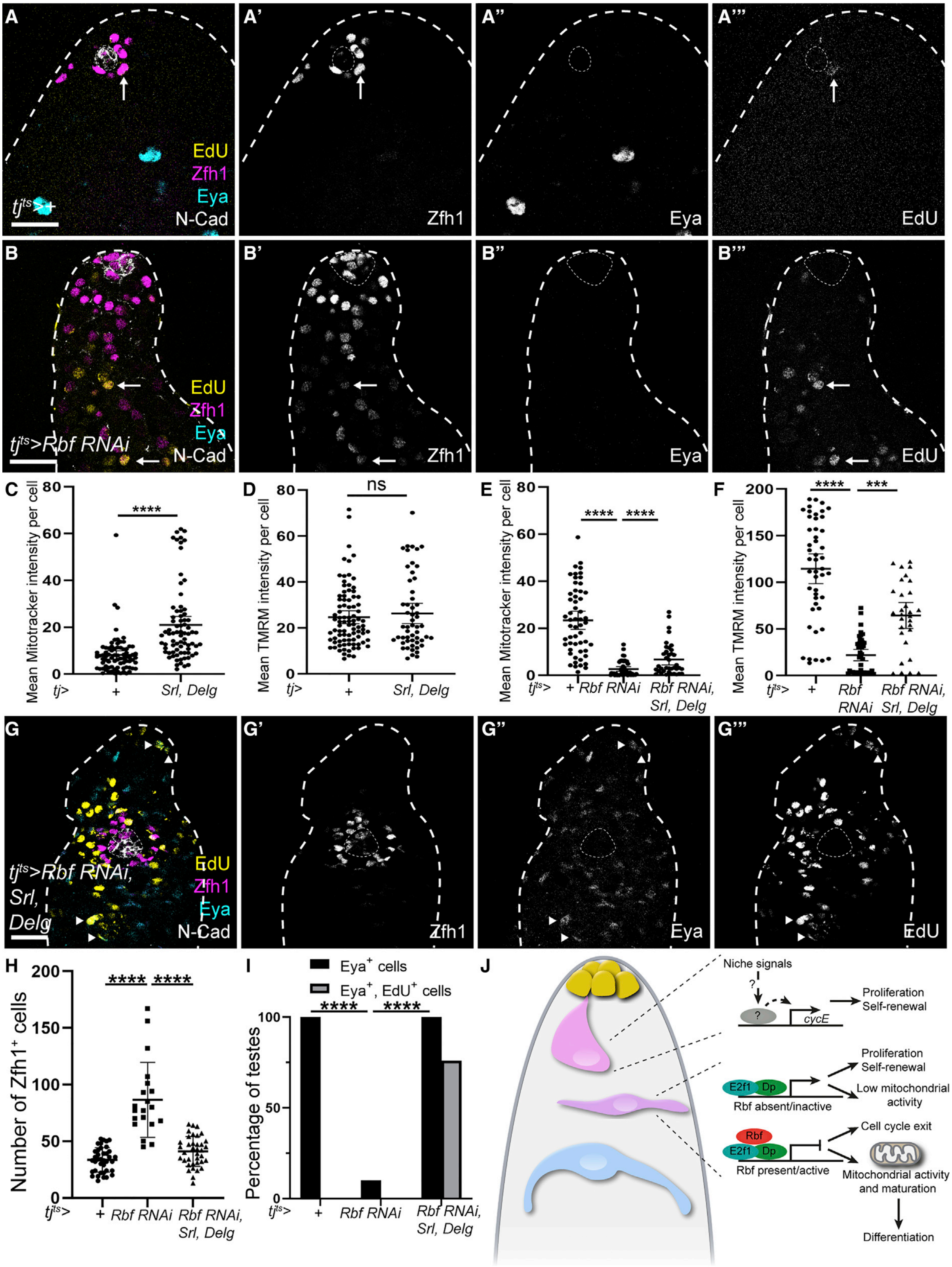

(legend on next page)

test). We confirmed that Rbf protein was absent in the somatic lineage in these rescued testes (Figure S7C). Similarly, clones expressing Rbf RNAi together with either Srl or Delg were partially rescued in their ability to differentiate (Figures S7D–S7H), and contained Eya-expressing cyst cells in 47% (N = 36) and 26% (N = 35) of cases, respectively, compared with 9% in Rbf knockdown alone (N = 46). Although the increase of differentiated cells in Delg-overexpressing clones was not significant (Fisher's exact test, $p = 0.06$), overexpression of Srl resulted in a statistically significant rescue of differentiation (Fisher's exact test, $p < 0.0001$).

We observed that the rescued testes did not appear completely normal. Compared with controls, the rescued Eya-positive cells appeared halted in their differentiation: they did not express as high levels of Eya and their nuclei did not grow as large as those of wild-type differentiated cyst cells (compare Figures 7A and 7G). Importantly, when we assayed for proliferation in the rescued testes, we observed Eya-positive, Zfh1-negative cells that were also EdU positive (Figure 7G, arrowheads) in 77% of testes (N = 43), which we never observed in controls (Figure 7I, N = 16, $p < 0.0005$, Fisher's exact test).

Thus, increasing mitochondrial biogenesis rescues the ability of Rbf-deficient CySCs to differentiate but not to exit the cell cycle. These data suggest that coordinating cell-cycle exit and differentiation in cyst cells is achieved by Rbf-dependent inhibition of E2f1/Dp activity, enabling a metabolic state that permits differentiation.

DISCUSSION

Our experiments show that Rbf coordinates differentiation and cell-cycle exit in G1 by silencing E2f1/Dp activity and enabling a metabolic state compatible with differentiation (Figure 7J).

Our observations concur with previous studies showing that several regulatory networks control S phase entry in *Drosophila* (Duronio et al., 1998; Frolov et al., 2001; Royzman et al., 1997; Zappia and Frolov, 2016). In CySCs, while *CycE* is necessary for proliferation, *E2f1* and *Dp* are dispensable. *CycE* expression is thought to be transcriptionally induced by E2f1/Dp and we note that ectopic E2f1/Dp is able to induce *CycE* expression, as *CycE* transcripts were upregulated in Rbf-deficient testes (Figure 4B), and ectopic CycE was observed in larval testes mutant for *Rbf* (Dominado et al., 2016). However, continued proliferation and CycE expression in *Dp* or *E2f1* mutant CySCs indicates that other inputs impinge on *CycE* regulation. One likely possibility is that self-renewal signals induce *CycE* expression and, indeed, two signals known to be active in CySCs and required for their self-renewal, Hedgehog and Hippo, are known regulators of *CycE* (Amoyel et al., 2013, 2014; Duman-Scheel et al., 2002; Huang et al., 2005; Michel et al., 2012). In particular, Zfh1 directly inhibits Hippo activity in CySCs, restricting Yki activation to the CySC pool, and suggesting a possible link to *cycE* expression (Albert et al., 2018). It is intriguing to note that, in female GSCs, CycE is detected in G2 and M phases as well as G1, indicating that its expression may be regulated differently in different cell types (Hsu et al., 2008).

Despite a reporter pattern consistent with periodic cell-cycle-dependent activation, E2f1/Dp are not required for normal cycling in CySCs, consistent with findings that cells lacking E2f/Dp activity both in the *Drosophila* embryo and larva can express cell-cycle genes and continue to proliferate (Duronio et al., 1998; Frolov et al., 2001; Royzman et al., 1997). Indeed, recent work showed that *Dp* null mutants could be rescued to adulthood if Dp was restored only in muscle (Zappia and Frolov, 2016). Similarly, mouse retinal progenitors and Müller glia deficient for all three mammalian activator E2fs could continue to proliferate (Chen et al., 2009). Thus, other factors are capable of controlling expression of the genes required for DNA replication, at least partly redundantly with the E2f complex. Several transcription factors have been described which have overlapping targets with E2f/Dp, including DREF and, recently, the SP/Krüppel-like factor Cabut (Cbt) (Tue et al., 2017; Zhang et al., 2021). Intriguingly, Cbt can drive PCNA expression, consistent with our observation that PCNA-GFP is reduced but not absent upon Dp loss of function (Figures 2E and S1D–S1F). Thus, continued proliferation and expression of replication genes in the absence of E2f1/Dp could be due either to de-repression from a lack of E2f repressive activity, or to active regulation by other factors such as Cbt. Nonetheless, since Cbt does not drive *CycE* expression (Zhang et al., 2021), it seems likely that a combination of regulators of replication genes together with specific regulators of *CycE* are required to promote cell-cycle entry and progression in CySCs.

Our data instead argue that the role of E2f1/Dp activity is to promote a metabolic state that prevents differentiation. Thus, restraining E2f1/Dp activity through Rbf is essential to allow cyst

Figure 7. Promoting mitochondrial biogenesis can rescue differentiation but not cell-cycle exit in Rbf knockdown testes

(A, B, and G) Testes from control (A), Rbf knockdown (B), or Rbf knockdown together with Srl and Delg expression (G) labeled with Zfh1 (magenta) (A', B', and G'), Eya (cyan) (A'', B'', and G''), EdU (yellow) (A''', B''', and G'''), and N-Cadherin (N-Cad, white).

(C) Mean intensity of MitoTracker fluorescence per CySC in control and *tj > Srl*, *Delg* testes. **** $p < 0.0001$ determined by Mann-Whitney test.

(D) Mean intensity of TMRM fluorescence per CySC in control and *tj > Srl*, *Delg* testes. No significant differences were observed.

(E) Mean intensity of MitoTracker fluorescence per CySC in control, Rbf-deficient, and tj^{ts} > *Rbf RNAi*, *Srl*, *Delg* CySCs. ****$p < 0.0001$ determined by Mann-Whitney test.

(F) Mean intensity of TMRM fluorescence per CySC in control, Rbf-deficient, and tj^{ts} > *Rbf RNAi*, *Srl*, *Delg* testes. ****$p < 0.0001$ determined by Mann-Whitney test.

(G) Srl and Delg expression in Rbf knockdowns restores Eya expression and restricts Zfh1 around the hub. EdU$^+$, Eya$^+$ cells (arrowheads) are visible away from the hub. Dotted lines outline the hub. Scale bars, 20 μm.

(H) Number of Zfh1$^+$ cells in control, Rbf knockdown and Rbf knockdown together with Delg and Srl overexpression. ****$p < 0.0001$ as determined by Kruskal-Wallis and Dunn's multiple comparisons test.

(I) Fraction of testes containing Eya$^+$ cyst cells (black bars) and Eya$^+$, EdU$^+$ cells (gray bars). N = 38 for controls, N = 20 for Rbf RNAi, and N = 37 for Rbf RNAi, UAS-Srl, and UAS-Delg.

(J) Diagram summarizing the regulation of cell-cycle progression and differentiation by Rbf in CySCs.

cell differentiation, such that both differentiation and cell-cycle exit are coordinated through regulation of E2f/Dp. In the testis, the critical window in which E2f1/Dp activity impacts cell identity is not in the CySCs themselves, but in their daughter cells that are leaving the niche and initiating differentiation (Figure 7J).

Rb and E2f/Dp regulate metabolism in mice and *Drosophila* (Blanchet et al., 2011; Guarner et al., 2017; Nicolay et al., 2015; Sankaran et al., 2008; Zappia et al., 2019). Intriguingly, Rbf, E2f1, E2f2, and Dp directly bind the enhancers of several genes encoding mitochondrial-associated proteins both in *Drosophila* larvae and mammalian cells (Ambrus et al., 2013; Blanchet et al., 2011). However, the mechanisms of action appear cell specific as in larval *Drosophila* tissues E2f and Dp maintain mitochondrial gene expression and activity, while in differentiated skeletal muscle E2f1 acts together with Rb to inhibit oxidative metabolic gene expression.

Many studies have shown a critical role for mitochondria in stem cell differentiation, both in *Drosophila* and mammalian tissues (Chakrabarty and Chandel, 2021; Schell et al., 2017; Senos Demarco and Jones, 2019; Teixeira et al., 2015). Several mechanisms have been proposed by which this action of mitochondria on cell identity could be mediated, from ROS production to impacts on histone marks through metabolic intermediates (Chakrabarty and Chandel, 2021; Tatapudy et al., 2017). What controls the changes in mitochondrial activity during differentiation is still poorly understood. Indeed, our results show that increasing mitochondrial mass does not result in increased mitochondrial activity, indicating that activity is regulated independently of biogenesis. The rounded and immature appearance of mitochondria in CySCs lacking Rbf suggests that biogenesis and/or fusion dynamics may be responsible for the decreased activity, rather than a reduced availability of fuel for mitochondrial oxidation. Alternatively, an intriguing interpretation of our results is that differentiation depends on the number of mitochondria per cell, and that the rescues we observe are simply a consequence of increasing mitochondrial mass rather than activity. Future work will determine the mechanisms by which mitochondrial numbers and/or activity change in coordination with other factors known to control cell identity to promote differentiation.

Overall, our results suggest a model for how cell-cycle exit and differentiation are linked in CySCs: by limiting the ability of cells to change their metabolic state, E2f1/Dp activity ensures that cycling cells cannot differentiate.

Limitations of the study

The design of our experiments using *tj-Gal4* to sort cells means that our sequencing approach identified both likely Dp/E2f1 transcriptional targets and indirect targets that are upregulated as a consequence of an increase in the representation of CySC-like cells in the samples. Nonetheless, we validated that *Ald1* expression was downregulated in CySCs, indicating that at least some of the genes identified were indeed affected in CySCs themselves.

In CySCs, many genes encoding mitochondrial factors have reduced expression upon ectopic E2f1/Dp activity in a manner antagonized by Rbf. These observations suggest that in the testis the regulation may be indirect. In many instances, Rb loss results in dysregulation of chromatin regulators, suggesting a potential mechanism by which these effects could be mediated (Benevolenskaya et al., 2005; Gonzalo et al., 2005).

STAR★METHODS

Detailed methods are provided in the online version of this paper and include the following:

- KEY RESOURCES TABLE
- RESOURCE AVAILABILITY
  - Lead contact
  - Materials availability
  - Data and code availability
- EXPERIMENTAL MODEL AND SUBJECT DETAILS
  - Fly stocks and husbandry
- METHOD DETAILS
  - Immunohistochemistry
  - RNAseq experiments
  - Imaging of mitochondria
  - Electron microscopy
- QUANTIFICATION AND STATISTICAL ANALYSIS

SUPPLEMENTAL INFORMATION

Supplemental information can be found online at https://doi.org/10.1016/j.celrep.2022.110774.

ACKNOWLEDGMENTS

We thank members of the fly community for fly stocks and reagents, Andrew Herman and Lorena Sueiro Ballesteros at the University of Bristol and Gavin Giel and the Flow Cytometry Core Facility at Philipps University Marburg for FACS, Christy Waterfall and the Bristol Genomics Facility and Andreas Dahl and Susanne Reinhardt at the CRTD Deep Sequencing Core Facility for the RNA sequencing. Sabina Huhn and Ljubinka Cigoja provided essential technical assistance in fly genetics, molecular cloning, and immunohistochemistry. Many thanks to members of the Fernandes, Poole, and Barrios labs for constructive discussions, and to Alex Gould, Parthive Patel, and Vilaiwan Fernandes as well as the anonymous reviewers for critical feedback on the manuscript. This work was supported by an MRC Career Development Award MR/P009646/2, a UCL WISSF Flexible Support Award, and a UKRI Covid Allowance to M.A. and a DFG grant BO 3270/3-1 to C.B.

AUTHOR CONTRIBUTIONS

D.S.d.l.M., S.H.-M., L.P., C.B., and M.A. conducted the experiments and analyzed the data. Experiments were conceptualized and designed by D.S.d.l.M., M.A., and C.B. D.S.d.l.M. and M.A. wrote the manuscript, with editing contributions from C.B. All authors contributed to the article and approved the submitted version.

DECLARATION OF INTERESTS

The authors declare no competing interests.

Received: February 22, 2021
Revised: February 21, 2022
Accepted: April 12, 2022
Published: May 10, 2022

REFERENCES

Ables, E.T., and Drummond-Barbosa, D. (2013). Cyclin E controls Drosophila female germline stem cell maintenance independently of its role in proliferation

by modulating responsiveness to niche signals. Development *140*, 530–540. https://doi.org/10.1242/dev.088583.

Albert, E.A., Puretskaia, O.A., Terekhanova, N.V., Labudina, A., and Bokel, C. (2018). Direct control of somatic stem cell proliferation factors by the Drosophila testis stem cell niche. Development *145*, dev156315.

Ambrus, A.M., Islam, A.B., Holmes, K.B., Moon, N.S., Lopez-Bigas, N., Benevolenskaya, E.V., and Frolov, M.V. (2013). Loss of dE2F compromises mitochondrial function. Dev. Cell *27*, 438–451. https://doi.org/10.1016/j.devcel.2013.10.002.

Amoyel, M., Anderson, J., Suisse, A., Glasner, J., and Bach, E.A. (2016). Socs36E controls niche competition by repressing MAPK signaling in the Drosophila testis. PLoS Genet. *12*, e1005815. https://doi.org/10.1371/journal.pgen.1005815.

Amoyel, M., Sanny, J., Burel, M., and Bach, E.A. (2013). Hedgehog is required for CySC self-renewal but does not contribute to the GSC niche in the Drosophila testis. Development *140*, 56–65. https://doi.org/10.1242/dev.086413.

Amoyel, M., Simons, B.D., and Bach, E.A. (2014). Neutral competition of stem cells is skewed by proliferative changes downstream of Hh and Hpo. EMBO J. *33*, 2295–2313. https://doi.org/10.15252/embj.201387500.

Benevolenskaya, E.V., Murray, H.L., Branton, P., Young, R.A., and Kaelin, W.G., Jr. (2005). Binding of pRB to the PHD protein RBP2 promotes cellular differentiation. Mol. Cell *18*, 623–635. https://doi.org/10.1016/j.molcel.2005.05.012.

Blanchet, E., Annicotte, J.S., Lagarrigue, S., Aguilar, V., Clape, C., Chavey, C., Fritz, V., Casas, F., Apparailly, F., Auwerx, J., and Fajas, L. (2011). E2F transcription factor-1 regulates oxidative metabolism. Nat. Cell Biol. *13*, 1146–1152. https://doi.org/10.1038/ncb2309.

Buttitta, L.A., and Edgar, B.A. (2007). Mechanisms controlling cell cycle exit upon terminal differentiation. Curr. Opin. Cell Biol. *19*, 697–704. https://doi.org/10.1016/j.ceb.2007.10.004.

Cappell, S.D., Chung, M., Jaimovich, A., Spencer, S.L., and Meyer, T. (2016). Irreversible APC(Cdh1) inactivation underlies the point of No return for cell-cycle entry. Cell *166*, 167–180. https://doi.org/10.1016/j.cell.2016.05.077.

Chakrabarty, R.P., and Chandel, N.S. (2021). Mitochondria as signaling organelles control mammalian stem cell fate. Cell Stem Cell *28*, 394–408. https://doi.org/10.1016/j.stem.2021.02.011.

Chen, D., Pacal, M., Wenzel, P., Knoepfler, P.S., Leone, G., and Bremner, R. (2009). Division and apoptosis of E2f-deficient retinal progenitors. Nature *462*, 925–929. https://doi.org/10.1038/nature08544.

Cheng, J., Tiyaboonchai, A., Yamashita, Y.M., and Hunt, A.J. (2011). Asymmetric division of cyst stem cells in Drosophila testis is ensured by anaphase spindle repositioning. Development *138*, 831–837. https://doi.org/10.1242/dev.057901.

Cheng, T., Rodrigues, N., Shen, H., Yang, Y.g., Dombkowski, D., Sykes, M., and Scadden, D.T. (2000). Hematopoietic stem cell quiescence maintained by p21cip1/waf1. Science *287*, 1804–1808. https://doi.org/10.1126/science.287.5459.1804.

Dimova, D.K., and Dyson, N.J. (2005). The E2F transcriptional network: old acquaintances with new faces. Oncogene *24*, 2810–2826. https://doi.org/10.1038/sj.onc.1208612.

Dimova, D.K., Stevaux, O., Frolov, M.V., and Dyson, N.J. (2003). Cell cycle-dependent and cell cycle-independent control of transcription by the Drosophila E2F/RB pathway. Genes Dev. *17*, 2308–2320. https://doi.org/10.1101/gad.1116703.

Doherty, C.A., Diegmiller, R., Kapasiawala, M., Gavis, E.R., and Shvartsman, S.Y. (2021). Coupled oscillators coordinate collective germline growth. Dev. Cell *56*, 860–870.e8. https://doi.org/10.1016/j.devcel.2021.02.015.

Dominado, N., La Marca, J.E., Siddall, N.A., Heaney, J., Tran, M., Cai, Y., Yu, F., Wang, H., Somers, W.G., Quinn, L.M., and Hime, G.R. (2016). Rbf regulates Drosophila spermatogenesis via control of somatic stem and progenitor cell fate in the larval testis. Stem Cell Rep. *7*, 1152–1163. https://doi.org/10.1016/j.stemcr.2016.11.007.

Dubey, P., Kapoor, T., Gupta, S., Shirolikar, S., and Ray, K. (2019). Atypical septate junctions maintain the somatic enclosure around maturing spermatids and prevent premature sperm release in Drosophila testis. Biol. Open *8*, bio036939. https://doi.org/10.1242/bio.036939.

Duman-Scheel, M., Weng, L., Xin, S., and Du, W. (2002). Hedgehog regulates cell growth and proliferation by inducing Cyclin D and Cyclin E. Nature *417*, 299–304. https://doi.org/10.1038/417299a.

Duronio, R.J., Bonnette, P.C., and O'Farrell, P.H. (1998). Mutations of the Drosophila dDP, dE2F, and cyclin E genes reveal distinct roles for the E2F-DP transcription factor and cyclin E during the G1-S transition. Mol. Cell Biol. *18*, 141–151. https://doi.org/10.1128/mcb.18.1.141.

Duronio, R.J., Brook, A., Dyson, N., and O'Farrell, P.H. (1996). E2F-induced S phase requires cyclin E. Genes Dev. *10*, 2505–2513. https://doi.org/10.1101/gad.10.19.2505.

Duronio, R.J., O'Farrell, P.H., Xie, J.E., Brook, A., and Dyson, N. (1995). The transcription factor E2F is required for S phase during Drosophila embryogenesis. Genes Dev. *9*, 1445–1455. https://doi.org/10.1101/gad.9.12.1445.

Dynlacht, B.D., Brook, A., Dembski, M., Yenush, L., and Dyson, N. (1994). DNA-binding and trans-activation properties of Drosophila E2F and DP proteins. Proc. Natl. Acad. Sci. U S A *91*, 6359–6363. https://doi.org/10.1073/pnas.91.14.6359.

Fabrizio, J.J., Boyle, M., and DiNardo, S. (2003). A somatic role for eyes absent (eya) and sine oculis (so) in Drosophila spermatocyte development. Dev. Biol. *258*, 117–128. https://doi.org/10.1016/s0012-1606(03)00127-1.

Fairchild, M.J., Islam, F., and Tanentzapf, G. (2017). Identification of genetic networks that act in the somatic cells of the testis to mediate the developmental program of spermatogenesis. PLoS Genet. *13*, e1007026. https://doi.org/10.1371/journal.pgen.1007026.

Fairchild, M.J., Smendziuk, C.M., and Tanentzapf, G. (2015). A somatic permeability barrier around the germline is essential for Drosophila spermatogenesis. Development *142*, 268–281. https://doi.org/10.1242/dev.114967.

Fairchild, M.J., Yang, L., Goodwin, K., and Tanentzapf, G. (2016). Occluding junctions maintain stem cell niche homeostasis in the fly testes. Curr. Biol. *26*, 2492–2499. https://doi.org/10.1016/j.cub.2016.07.012.

Flaherty, M.S., Salis, P., Evans, C.J., Ekas, L.A., Marouf, A., Zavadil, J., Banerjee, U., and Bach, E.A. (2010). Chinmo is a functional effector of the JAK/STAT pathway that regulates eye development, tumor formation, and stem cell self-renewal in Drosophila. Dev. Cell *18*, 556–568. https://doi.org/10.1016/j.devcel.2010.02.006.

Frolov, M.V., Huen, D.S., Stevaux, O., Dimova, D., Balczarek-Strang, K., Elsdon, M., and Dyson, N.J. (2001). Functional antagonism between E2F family members. Genes Dev. *15*, 2146–2160. https://doi.org/10.1101/gad.903901.

Frolov, M.V., Moon, N.S., and Dyson, N.J. (2005). dDP is needed for normal cell proliferation. Mol. Cell Biol. *25*, 3027–3039. https://doi.org/10.1128/mcb.25.8.3027-3039.2005.

Ge, S.X., Jung, D., and Yao, R. (2020). ShinyGO: a graphical gene-set enrichment tool for animals and plants. Bioinformatics *36*, 2628–2629. https://doi.org/10.1093/bioinformatics/btz931.

Gonczy, P., and DiNardo, S. (1996). The germ line regulates somatic cyst cell proliferation and fate during Drosophila spermatogenesis. Development *122*, 2437–2447. https://doi.org/10.1242/dev.122.8.2437.

Gonzalo, S., Garcia-Cao, M., Fraga, M.F., Schotta, G., Peters, A.H., Cotter, S.E., Eguia, R., Dean, D.C., Esteller, M., Jenuwein, T., et al. (2005). Role of the RB1 family in stabilizing histone methylation at constitutive heterochromatin. Nat. Cell Biol. *7*, 420–428. https://doi.org/10.1038/ncb1235.

Greenspan, L.J., and Matunis, E.L. (2018). Retinoblastoma intrinsically regulates niche cell quiescence, identity, and niche number in the adult Drosophila testis. Cell Rep. *24*, 3466–3476.e8. https://doi.org/10.1016/j.celrep.2018.08.083.

Guarner, A., Morris, R., Korenjak, M., Boukhali, M., Zappia, M.P., Van Rechem, C., Whetstine, J.R., Ramaswamy, S., Zou, L., Frolov, M.V., et al. (2017). E2F/DP prevents cell-cycle progression in endocycling fat body cells by

CellPress
OPEN ACCESS

suppressing dATM expression. Dev. Cell *43*, 689–703.e5. https://doi.org/10.1016/j.devcel.2017.11.008.

Hardy, R.W., Tokuyasu, K.T., Lindsley, D.L., and Garavito, M. (1979). The germinal proliferation center in the testis of Drosophila melanogaster. J. Ultrastruct. Res. *69*, 180–190. https://doi.org/10.1016/s0022-5320(79)90108-4.

Herrera, S.C., Sainz de la Maza, D., Grmai, L., Margolis, S., Plessel, R., Burel, M., O'Connor, M., Amoyel, M., and Bach, E.A. (2021). Proliferative stem cells maintain quiescence of their niche by secreting the Activin inhibitor Follistatin. Dev. Cell *56*, 2284–2294.e6. https://doi.org/10.1016/j.devcel.2021.07.010.

Hsu, H.J., LaFever, L., and Drummond-Barbosa, D. (2008). Diet controls normal and tumorous germline stem cells via insulin-dependent and -independent mechanisms in Drosophila. Dev. Biol. *313*, 700–712. https://doi.org/10.1016/j.ydbio.2007.11.006.

Huang, J., Wu, S., Barrera, J., Matthews, K., and Pan, D. (2005). The Hippo signaling pathway coordinately regulates cell proliferation and apoptosis by inactivating Yorkie, the Drosophila Homolog of YAP. Cell *122*, 421–434. https://doi.org/10.1016/j.cell.2005.06.007.

Inaba, M., Yuan, H., and Yamashita, Y.M. (2011). String (Cdc25) regulates stem cell maintenance, proliferation and aging in Drosophila testis. Development *138*, 5079–5086. https://doi.org/10.1242/dev.072579.

Ishida, S., Huang, E., Zuzan, H., Spang, R., Leone, G., West, M., and Nevins, J.R. (2001). Role for E2F in control of both DNA replication and mitotic functions as revealed from DNA microarray analysis. Mol. Cell Biol. *21*, 4684–4699. https://doi.org/10.1128/mcb.21.14.4684-4699.2001.

Jones, D.L., and Wagers, A.J. (2008). No place like home: anatomy and function of the stem cell niche. Nat. Rev. Mol. Cell Biol. *9*, 11–21. https://doi.org/10.1038/nrm2319.

Kiger, A.A., White-Cooper, H., and Fuller, M.T. (2000). Somatic support cells restrict germline stem cell self-renewal and promote differentiation. Nature *407*, 750–754. https://doi.org/10.1038/35037606.

Kim, D., Paggi, J.M., Park, C., Bennett, C., and Salzberg, S.L. (2019). Graph-based genome alignment and genotyping with HISAT2 and HISAT-genotype. Nat. Biotechnol. *37*, 907–915.

Korenjak, M., Anderssen, E., Ramaswamy, S., Whetstine, J.R., and Dyson, N.J. (2012). RBF binding to both canonical E2F targets and noncanonical targets depends on functional dE2F/dDP complexes. Mol. Cell Biol. *32*, 4375–4387. https://doi.org/10.1128/mcb.00536-12.

Kucukural, A., Yukselen, O., Ozata, D.M., Moore, M.J., and Garber, M. (2019). DEBrowser: interactive differential expression analysis and visualization tool for count data. BMC Genom. *20*, 6. https://doi.org/10.1186/s12864-018-5362-x.

Laker, R.C., Xu, P., Ryall, K.A., Sujkowski, A., Kenwood, B.M., Chain, K.H., Zhang, M., Royal, M.A., Hoehn, K.L., Driscoll, M., et al. (2014). A novel MitoTimer reporter gene for mitochondrial content, structure, stress, and damage in vivo. J. Biol. Chem. *289*, 12005–12015. https://doi.org/10.1074/jbc.m113.530527.

Leatherman, J.L., and Dinardo, S. (2008). Zfh-1 controls somatic stem cell self-renewal in the Drosophila testis and nonautonomously influences germline stem cell self-renewal. Cell Stem Cell *3*, 44–54. https://doi.org/10.1016/j.stem.2008.05.001.

Lee, T., and Luo, L. (2001). Mosaic analysis with a repressible cell marker (MARCM) for Drosophila neural development. Trends Neurosci. *24*, 251–254. https://doi.org/10.1016/s0166-2236(00)01791-4.

Li, M.A., Alls, J.D., Avancini, R.M., Koo, K., and Godt, D. (2003). The large Maf factor Traffic Jam controls gonad morphogenesis in Drosophila. Nat. Cell Biol. *5*, 994–1000. https://doi.org/10.1038/ncb1058.

Lin, C.C., and Potter, C.J. (2016). Editing transgenic DNA components by inducible gene replacement in Drosophila melanogaster. Genetics *203*, 1613–1628. https://doi.org/10.1534/genetics.116.191783.

Love, M.I., Huber, W., and Anders, S. (2014). Moderated estimation of fold change and dispersion for RNA-seq data with DESeq2. Genome Biol. *15*, 550.

McGuire, S.E., Mao, Z., and Davis, R.L. (2004). Spatiotemporal gene expression targeting with the TARGET and gene-switch systems in Drosophila. Sci. STKE *2004*, pl6. https://doi.org/10.1126/stke.2202004pl6.

Merrill, R.A., Flippo, K.H., and Strack, S. (2017). Measuring mitochondrial shape with ImageJ. In Techniques to Investigate Mitochondrial Function in Neurons, S. Strack and Y.M. Usachev, eds. (Springer), pp. 31–48.

Michel, M., Kupinski, A.P., Raabe, I., and Bokel, C. (2012). Hh signalling is essential for somatic stem cell maintenance in the Drosophila testis niche. Development *139*, 2663–2669. https://doi.org/10.1242/dev.075242.

Michel, M., Raabe, I., Kupinski, A.P., Perez-Palencia, R., and Bokel, C. (2011). Local BMP receptor activation at adherens junctions in the Drosophila germline stem cell niche. Nat. Commun. *2*, 415. https://doi.org/10.1038/ncomms1426.

Narasimha, A.M., Kaulich, M., Shapiro, G.S., Choi, Y.J., Sicinski, P., and Dowdy, S.F. (2014). Cyclin D activates the Rb tumor suppressor by mono-phosphorylation. Elife *3*, e02872. https://doi.org/10.7554/elife.02872.

Nicolay, B.N., Danielian, P.S., Kottakis, F., Lapek, J.D., Jr., Sanidas, I., Miles, W.O., Dehnad, M., Tschop, K., Gierut, J.J., Manning, A.L., et al. (2015). Proteomic analysis of pRb loss highlights a signature of decreased mitochondrial oxidative phosphorylation. Genes Dev. *29*, 1875–1889. https://doi.org/10.1101/gad.264127.115.

Papagiannouli, F., Berry, C.W., and Fuller, M.T. (2019). The Dlg module and clathrin-mediated endocytosis regulate EGFR signaling and cyst cell-germline coordination in the Drosophila testis. Stem Cell Rep. *12*, 1024–1040. https://doi.org/10.1016/j.stemcr.2019.03.008.

Pennycook, B.R., and Barr, A.R. (2020). Restriction point regulation at the crossroads between quiescence and cell proliferation. FEBS Lett. *594*, 2046–2060. https://doi.org/10.1002/1873-3468.13867.

Pertea, M., Pertea, G.M., Antonescu, C.M., Chang, T.-C., Mendell, J.T., and Salzberg, S.L. (2015). StringTie enables improved reconstruction of a transcriptome from RNA-seq reads. Nat. Biotechnol. *33*, 290–295.

Rera, M., Bahadorani, S., Cho, J., Koehler, C.L., Ulgherait, M., Hur, J.H., Ansari, W.S., Lo, T., Jr., Jones, D.L., and Walker, D.W. (2011). Modulation of longevity and tissue homeostasis by the Drosophila PGC-1 homolog. Cell Metab. *14*, 623–634. https://doi.org/10.1016/j.cmet.2011.09.013.

Royzman, I., Whittaker, A.J., and Orr-Weaver, T.L. (1997). Mutations in Drosophila DP and E2F distinguish G1-S progression from an associated transcriptional program. Genes Dev. *11*, 1999–2011. https://doi.org/10.1101/gad.11.15.1999.

Rubin, S.M., Sage, J., and Skotheim, J.M. (2020). Integrating old and new paradigms of G1/S control. Mol. Cell *80*, 183–192. https://doi.org/10.1016/j.molcel.2020.08.020.

Ruijtenberg, S., and van den Heuvel, S. (2016). Coordinating cell proliferation and differentiation: antagonism between cell cycle regulators and cell type-specific gene expression. Cell Cycle *15*, 196–212. https://doi.org/10.1080/15384101.2015.1120925.

Sage, J. (2012). The retinoblastoma tumor suppressor and stem cell biology. Genes Dev. *26*, 1409–1420. https://doi.org/10.1101/gad.193730.112.

Sankaran, V.G., Orkin, S.H., and Walkley, C.R. (2008). Rb intrinsically promotes erythropoiesis by coupling cell cycle exit with mitochondrial biogenesis. Genes Dev. *22*, 463–475. https://doi.org/10.1101/gad.1627208.

Sawado, T., Yamaguchi, M., Nishimoto, Y., Ohno, K., Sakaguchi, K., and Matsukage, A. (1998). dE2F2, a novel E2F-family transcription factor in Drosophila melanogaster. Biochem. Biophys. Res. Commun. *251*, 409–415. https://doi.org/10.1006/bbrc.1998.9407.

Schell, J.C., Wisidagama, D.R., Bensard, C., Zhao, H., Wei, P., Tanner, J., Flores, A., Mohlman, J., Sorensen, L.K., Earl, C.S., et al. (2017). Control of intestinal stem cell function and proliferation by mitochondrial pyruvate metabolism. Nat. Cell Biol. *19*, 1027–1036. https://doi.org/10.1038/ncb3593.

Schneider, C.A., Rasband, W.S., and Eliceiri, K.W. (2012). NIH Image to ImageJ: 25 years of image analysis. Nat. Methods *9*, 671–675.

Schwarz, C., Johnson, A., Koivomagi, M., Zatulovskiy, E., Kravitz, C.J., Doncic, A., and Skotheim, J.M. (2018). A precise Cdk activity threshold determines

passage through the restriction point. Mol. Cell *69*, 253–264.e5. https://doi.org/10.1016/j.molcel.2017.12.017.

Senos Demarco, R., and Jones, D.L. (2019). Mitochondrial fission regulates germ cell differentiation by suppressing ROS-mediated activation of Epidermal Growth Factor Signaling in the Drosophila larval testis. Sci. Rep. *9*, 19695. https://doi.org/10.1038/s41598-019-55728-0.

Soufi, A., and Dalton, S. (2016). Cycling through developmental decisions: how cell cycle dynamics control pluripotency, differentiation and reprogramming. Development *143*, 4301–4311. https://doi.org/10.1242/dev.142075.

Stevaux, O., and Dyson, N.J. (2002). A revised picture of the E2F transcriptional network and RB function. Curr. Opin. Cell Biol. *14*, 684–691. https://doi.org/10.1016/s0955-0674(02)00388-5.

Tatapudy, S., Aloisio, F., Barber, D., and Nystul, T. (2017). Cell fate decisions: emerging roles for metabolic signals and cell morphology. EMBO Rep. *18*, 2105–2118. https://doi.org/10.15252/embr.201744816.

Teixeira, F.K., Sanchez, C.G., Hurd, T.R., Seifert, J.R.K., Czech, B., Preall, J.B., Hannon, G.J., and Lehmann, R. (2015). ATP synthase promotes germ cell differentiation independent of oxidative phosphorylation. Nat. Cell Biol. *17*, 689–696. https://doi.org/10.1038/ncb3165.

Thacker, S.A., Bonnette, P.C., and Duronio, R.J. (2003). The contribution of E2F-regulated transcription to Drosophila PCNA gene function. Curr. Biol. *13*, 53–58. https://doi.org/10.1016/s0960-9822(02)01400-8.

Tiefenbock, S.K., Baltzer, C., Egli, N.A., and Frei, C. (2010). The Drosophila PGC-1 homologue Spargel coordinates mitochondrial activity to insulin signalling. EMBO J. *29*, 171–183. https://doi.org/10.1038/emboj.2009.330.

Tran, J., Brenner, T.J., and DiNardo, S. (2000). Somatic control over the germline stem cell lineage during Drosophila spermatogenesis. Nature *407*, 754–757. https://doi.org/10.1038/35037613.

Tue, N.T., Yoshioka, Y., Mizoguchi, M., Yoshida, H., Zurita, M., and Yamaguchi, M. (2017). DREF plays multiple roles during Drosophila development. Biochim. Biophys. Acta Gene Regul. Mech. *1860*, 705–712. https://doi.org/10.1016/j.bbagrm.2017.03.004.

Vara, C., Paytuvi-Gallart, A., Cuartero, Y., Le Dily, F., Garcia, F., Salva-Castro, J., Gomez, H.L., Julia, E., Moutinho, C., Aiese Cigliano, R., et al. (2019). Three-dimensional genomic structure and cohesin occupancy correlate with transcriptional activity during spermatogenesis. Cell Rep. *28*, 352–367.e9. https://doi.org/10.1016/j.celrep.2019.06.037.

Venken, K.J.T., Popodi, E., Holtzman, S.L., Schulze, K.L., Park, S., Carlson, J.W., Hoskins, R.A., Bellen, H.J., and Kaufman, T.C. (2010). A molecularly defined duplication set for the X chromosome of Drosophila melanogaster. Genetics *186*, 1111–1125. https://doi.org/10.1534/genetics.110.121285.

Vermeulen, K., Van Bockstaele, D.R., and Berneman, Z.N. (2003). The cell cycle: a review of regulation, deregulation and therapeutic targets in cancer. Cell Prolif *36*, 131–149. https://doi.org/10.1046/j.1365-2184.2003.00266.x.

Voog, J., Sandall, S.L., Hime, G.R., Resende, L.P., Loza-Coll, M., Aslanian, A., Yates, J.R., 3rd, Hunter, T., Fuller, M.T., and Jones, D.L. (2014). Escargot restricts niche cell to stem cell conversion in the Drosophila testis. Cell Rep. *7*, 722–734. https://doi.org/10.1016/j.celrep.2014.04.025.

Wang, Z., and Lin, H. (2005). The division of Drosophila germline stem cells and their precursors requires a specific cyclin. Curr. Biol. *15*, 328–333. https://doi.org/10.1016/j.cub.2005.02.016.

Wang, Z.A., and Kalderon, D. (2009). Cyclin E-dependent protein kinase activity regulates niche retention of Drosophila ovarian follicle stem cells. Proc. Natl. Acad. Sci. U S A *106*, 21701–21706. https://doi.org/10.1073/pnas.0909272106.

Yamaguchi, M., Hayashi, Y., and Matsukage, A. (1995). Essential role of E2F recognition sites in regulation of the proliferating cell nuclear antigen gene promoter during Drosophila development. J. Biol. Chem. *270*, 25159–25165. https://doi.org/10.1074/jbc.270.42.25159.

Yu, G., Wang, L.G., Han, Y., and He, Q.Y. (2012). clusterProfiler: an R package for comparing biological themes among gene clusters. OMICS *16*, 284–287. https://doi.org/10.1089/omi.2011.0118.

Zappia, M.P., and Frolov, M.V. (2016). E2F function in muscle growth is necessary and sufficient for viability in Drosophila. Nat. Commun. *7*, 10509. https://doi.org/10.1038/ncomms10509.

Zappia, M.P., Rogers, A., Islam, A.B., and Frolov, M.V. (2019). Rbf activates the myogenic transcriptional program to promote skeletal muscle differentiation. Cell Rep. *26*, 702–719.e6. https://doi.org/10.1016/j.celrep.2018.12.080.

Zhang, P., Katzaroff, A.J., Buttitta, L.A., Ma, Y., Jiang, H., Nickerson, D.W., Ovrebo, J.I., and Edgar, B.A. (2021). The Kruppel-like factor Cabut has cell cycle regulatory properties similar to E2F1. Proc. Natl. Acad. Sci. U S A *118*, e2015675118. https://doi.org/10.1073/pnas.2015675118.

STAR★METHODS

KEY RESOURCES TABLE

REAGENT or RESOURCE	SOURCE	IDENTIFIER
**Antibodies**		
Mouse monoclonal anti-Eya (1:20)	Developmental Studies Hybridoma Bank (DSHB)	Cat# eya10H6; RRID:AB_528232
Mouse monoclonal anti-Fas3 (1:20)	DSHB	Cat# 7G10; RRID:AB_528238
Chicken polyclonal anti-GFP (1:500)	Aves Lab	Cat# GFP-1010; RRID:AB_2307313
Rabbit polyclonal anti-GFP (1:500)	ThermoFisher	Cat# A6455; RRID:AB_221570
Rat monoclonal anti-NCad (1:20)	DSHB	Cat# MNCD2; RRID:AB_528119
Mouse monoclonal anti-Wg (1:200)	DSHB	Cat# 4D4; RRID:AB_528512
Mouse monoclonal anti-Rbf (1:15)	Gift of N. Dyson	RRID:AB_2567501
Mouse monoclonal anti-Dp clone Yun6 (1:5)	Gift of N. Dyson	RRID:AB_2889822
Guinea pig polyclonal anti-CycE (1:100)	Gift of J. Nordman	N/A
Rat polyclonal anti-Chinmo (1:50)	Gift of N.Sokol	RRID:AB_2570149
Guinea pig polyclonal anti-Tj (1:3000)	Gift of D. Godt	RRID:AB_2568583
Rabbit polyclonal anti-Zfh1 (1:5000)	Gift of R. Lehmann	N/A
**Chemicals, peptides, and recombinant proteins**		
Schneider's *Drosophila* medium	Merck	S0146
Tetramethylrhodamine (TMRM)	ThermoFisher	T668; CAS 115532-50-8
Mitotracker Red CMXRos	Cell Signaling	9082
5-ethynyl-2′-deoxyuridine (EdU)	Abcam	ab146186
**Critical commercial assays**		
SMART-Seq v4 Ultra	Takara Bio	R400752
Nextera XT DNA preparation kit	Illumina	FC-131-1024
**Deposited data**		
Rbf vs control RNA seq	This paper	UCL Data Repository: https://doi.org/10.5522/04/13484814
CySC vs cyst cell RNA seq	This paper	NCBI: PRJNA630200
**Experimental models: Organisms/strains**		
D. melanogaster: tj-Gal4	Amoyel lab	Flybase: FBti0034540
D. melanogaster: tub-Gal80^{ts}	Amoyel lab	Flybase: FBti0027797
D. melanogaster: tub-Gal80^{ts}	Amoyel lab	Flybase: FBti0027796
D. melanogaster: Zfh1-T2A-Gal4	Bökel lab	Flybase: FBal0340857
D. melanogaster: Zfh1-T2A-Gal80	This study	N/A
D. melanogaster: Dp RNAi: P{GD4444}	Vienna Drosophila Resource Center (VDRC)	VDRC: 12722; Flybase: FBti0091763
D. melanogaster: UAS-E2f1, UAS-Dp	Laura Buttitta	N/A
D. melanogaster: w; P{UAS-CycE.L}ML1*	Bloomington Drosophila Stock Center (BDSC)	BDSC: 4781; Flybase: FBti0012496
D. melanogaster: Rbf RNAi: P{TRiP.GL01293}attP40	BDSC	BDSC 41863; Flybase: FBti0149374
D. melanogaster: Rbf RNAi: P{TRiP.HMS03004}attP2/TM3	BDSC	BDSC 36744; Flybase: FBti0146791
D. melanogaster: PCNA-GFP	Laura Buttitta	Flybase: FBti0210496
D. melanogaster: w1118; PBac{768.FSVS-0}Ald1CPTI002230	Kyoto Stock Center	Kyoto 115279; Flybase: FBti0143752
D. melanogaster: TI{TI}CycE^{sfGFP} (cycE-GFP)	Caroline Doherty and Stanislav Shvartsman	Flybase: FBal0367148
D. melanogaster: w1118; P{UAS-MitoTimer}3	BDSC	BDSC 57323; Flybase: FBti0161163
D. melanogaster: UAS-Srl	Hugo Stocker	N/A

(*Continued on next page*)

Continued

REAGENT or RESOURCE	SOURCE	IDENTIFIER
D. melanogaster: UAS-Delg	Martine Simonelig	N/A
D. melanogaster: P{PTT-un}P01986 (esg-GFP)	Leanne Jones	Flybase: FBti0196571
D. melanogaster: Dp(1;3)DC012	BDSC	BDSC 30222; Flybase: FBab0046286
D. melanogaster: Rbf^{14}	BDSC	BDSC 7435; Flybase: FBal0095620
D. melanogaster: $CycE^{AR95}$, FRT^{40A}	Agnes Audibert	Flybase: FBal0033578
D. melanogaster: $CycE^{WX}$, FRT^{40A}	Daniel Kalderon	Flybase: FBal0241968
D. melanogaster: FRT^{42D}, Dp^{a3}	Maxim Frolov	Flybase: FBal0063497
D. melanogaster: FRT^{42D}, Dp^{a4}	Maxim Frolov	Flybase: FBal0063496
D. melanogaster: FRT82B, E2f1rM729	BDSC	BDSC 35849; Flybase: FBti0003853
D. melanogaster: Df(2R)Exel7124/CyO	BDSC	BDSC 7872; Flybase: FBab0038034
D. melanogaster: Df(3R)Exel6186, P{XP-U}Exel6186/TM6B, Tb1	BDSC	BDSC 7665; Flybase: FBab0038241
Software and algorithms		
Image J	(Schneider et al., 2012)	https://imagej.nih.gov/ij/
RStudio	RStudio Team, 2020	http://www.rstudio.com/
clusterProfiler (R package) v3.14.3	Yu et al., 2012	https://guangchuangyu.github.io/software/clusterProfiler
DEBrowser	Kucukural et al., 2019	https://www.bioconductor.org/packages/release/bioc/html/debrowser.html
ShinyGO v0.75	Ge et al., 2020	http://bioinformatics.sdstate.edu/go/
A.I.R. RNAseq web-based analysis package	Vara et al., 2019	https://transcriptomics.sequentiabiotech.com/
Hisat2	(Kim et al., 2019)	http://daehwankimlab.github.io/hisat2/
StringTie	(Pertea et al., 2015)	https://ccb.jhu.edu/software/stringtie/
DESeq2	(Love et al., 2014)	https://bioconductor.org/packages/release/bioc/html/DESeq2.html

RESOURCE AVAILABILITY

Lead contact

Further information and requests for resources and reagents should be directed to and will be fulfilled by the lead contact, Marc Amoyel (marc.amoyel@ucl.ac.uk).

Materials availability

All Drosophila stocks generated in this study are available from the Lead Contact without restriction.

Data and code availability

RNA-seq data have been deposited on the UCL Research Data Repository for the Rbf experiment (https://doi.org/10.5522/04/13484814.v1) and on the NCBI Sequence Read Archive (NCBI: PRJNA630200) for the comparison of CySC and cyst cell transcriptomes.

This paper does not report original code.

Any additional information required to reanalyse the data reported in this paper is available from the lead contact upon request.

EXPERIMENTAL MODEL AND SUBJECT DETAILS

Fly stocks and husbandry

Lineage-wide misexpression and knockdown experiments were carried out using the *tj-Gal4* driver, together with a *Tub>Gal80*^{ts} transgene (referred to as *tj*^{ts}) to control the temporal pattern of expression (McGuire et al., 2004). Crosses were raised at 18°C. Males were collected 0-3 days after eclosion and shifted to 29°C for 10 days. The following stocks were used: *UAS-Rbf RNAi* (BDSC #41863 and #36744); *UAS-CycE* (BDSC #4781); *UAS-Dp RNAi* (VDRC #12722); *PCNA-GFP; UAS-E2f1, UAS-Dp* (gifts of L. Buttitta); *Ald1-GFP* (Kyoto DGRC #115279); *UAS-mitotimer* (BDSC #57323)*; UAS-Srl* (gift of H. Stocker); *UAS-Delg* (gift of M. Simonelig); *esg-GFP* (gift of L. Jones); *cycE-GFP* (gift of C. Doherty, S. Shvartsman and E. Gavis).

w;; zfh1-T2A-T2A-Gal80 (referred to in brief as *zfh1-Gal80*) was generated by crossing yw vasa-Cas9 first to zfh1-T2A-Gal4 w+/TM3, Sb (Albert et al., 2018) and then to the Pin/CyO; Gal4-Gal80Hack/TM6B (94E5) Gal4-Gal80 HACK stock (Lin and Potter, 2016) that contains all the required components such as gRNA genes, homology arms, and an eye RFP selection marker to insert a T2A-Gal80 cassette into the Gal4 ORF of any Gal4 transgene on the homologous chromosome.

For clonal analysis, flies were raised and maintained at 25°C. Adult flies were collected 0-3 days after eclosion and heat shocked at 37°C for 1 hour. Clonal CySCs were identified as Zfh1-positive cells adjacent to the hub that were also positive for the clone marker (GFP expression or absence of GFP or RFP, depending on the genotype). Negatively marked Rbf mutant clones were generated using a duplication of the X chromosome on the third chromosome, *Dp(1:3)DC012* (Venken et al., 2010), which fully rescues the viability of Rbf^{14} hemizygous mutants. The experimental genotype was Rbf^{14} *w/Y; hs-flp/+; ubi-GFP Dp(1:3)DC012* FRT^{2A}/FRT^{2A}. Control clones were generated in the same way but wild type for *Rbf* in the endogenous locus (*y,w,hsflp*^{122}*/Y;; ubi-GFP Dp(1:3)DC012* FRT^{2A}/FRT^{2A}).

Negatively-marked *E2f1* mutant clones were generated with FRT^{82B}*, ubi-RFP* (gift of E. Piddini). All other clones were generated by the MARCM technique (Lee and Luo, 2001). Stocks used to generate clones were: *y,w,hsflp*^{122}*,Tub>Gal4,UAS-nlsGFP;* FRT^{42D}*, Tub>Gal80,CD71; y,w,hsflp*^{122}*,Tub>Gal4,UAS-nlsGFP;*FRT^{40A}*,Tub>Gal80; y,w,hsflp*^{122}*,Tub>Gal4,UAS-nlsGFP;;* FRT^{82B}*,Tub>Gal80* and *w,hs-FLP,C587-Gal4,UAS-RedStinger;* FRT^{42D}*,Tub>Gal80*. We used the following alleles: $CycE^{AR95}$ (gift of A. Audibert); $E2f1^{rM729}$ (also known as $E2f1^{729}$, BDSC#35849); Dp^{a3} and Dp^{a4} (gift of M. Frolov). *Dp* and *Ef21* mutants were validated by lack of complementation against *Df(2R)Exel7124* (BDSC #7872), and *Df(3R)Exel6186* (BDSC #7665), respectively, and in addition, the Dp^{a3} hemizygous mutant was rescued to adulthood by Dp over-expression with *Mef2-Gal4*, as previously described (Zappia and Frolov, 2016).

METHOD DETAILS

Immunohistochemistry

The following antibodies were used: rat anti-Chinmo (gift of N.Sokol), 1:50; mouse anti-Eya (Developmental Studies Hybridoma Bank, DSHB), 1:20; mouse anti-Fas3 (DSHB), 1:20; mouse anti-Wg (DSHB), 1:500; chicken anti-GFP (Aves Lab, GFP-1010), 1:500; rabbit anti-GFP (Thermo Fisher, A6455), 1:500; rat anti-NCad (DSHB), 1:20; mouse anti-Rbf (gift of N. Dyson), 1:15; mouse anti-Dp (gift of N. Dyson), 1:5; guinea pig anti-Tj (gift of D. Godt), 1:3000; rabbit anti-Zfh1 (gift of R. Lehmann), 1:5000; guinea pig anti-CycE (gift of J. Nordman), 1:100. Fixing and immunohistochemistry was carried out as previously described (Flaherty et al., 2010; Michel et al., 2011). In brief, dissected abdomens were fixed in 4% paraformaldehyde in PBS for 15 minutes. Samples were washed twice in PBS, 0.5% Triton X-100 for 30 minutes then blocked in PBS, 1% BSA, 0.2% Triton X-100 (PBTB) for one hour, before overnight incubation in primary antibodies diluted in PBTB. Samples were then washed twice in PBTB for 30 minutes, and incubated in secondary antibodies diluted in PBTB for 2 hours at room temperature, then washed in PBS, 0.2% Triton X-100, and mounted on slides with Vectashield mounting medium for imaging.

For EdU staining, testes were dissected in Schneider's medium and incubated for 30 minutes at room temperature in Schneider's medium containing 10μM EdU. Samples were then fixed and incubated with primary and secondary antibodies as above. Click reaction was then carried out for 30 minutes at room temperature in the following reaction buffer: 2.5μM Alexa-405 picolyl azide (Click Chemistry Tools), 0.1 mM THPTA, 2 mM sodium ascorbate and 1 mM $CuSO_4$.

RNAseq experiments

CySCs and differentiating cyst cells were labelled with RedStinger expression driven by *zfh1-T2A-Gal4* and *tj-gal4; zfh1-Gal80,* respectively. In the case of Rbf knockdown experiment, somatic cells were labelled with GFP driven by *tj-gal4*. Testes were dissected in Schneider's medium and then separation buffer containing Schneider's medium, collagenase, trypsin and EDTA was added. Samples were vigorously agitated for 15-30 min. The resulting cell suspension was then filtered using a cell strainer and GFP- or RedStinger-positive cells were isolated by fluorescence-activated cell sorting. For sequencing of control CyCS and cyst cells, 150 cells per replica were sorted directly into a 384 well skirted plate (Eppendorf twintec 0030128648) containing per well 2 μL lysis buffer comprised of 1.9 μL of 0.2% Triton X- 100 diluted in nuclease free H2O (Invitrogen 10977049) and 0.1 μL 40 U/μL murine RNase Inhibitor (NEB M0314S). For the Rbf experiments, 10,000-15,000 cells were sorted into Eppendorf tubes containing Schneider's medium, prior to lysis.

mRNA was isolated from the samples, reverse transcribed, and amplified using the SmartSeq2 kit (Illumina) for the study within the somatic lineage and SMART-Seq v4 Ultra (Takara Bio) for the Rbf knockdown experiment. Libraries were then generated using the Nextera kit (Illumina) and 75bp single end read-sequencing was carried out.

Reads were quality checked and mapped using the A.I.R. RNAseq web-based analysis package (Sequentia Biotech, Barcelona). The underlying algorithms and software packages collated in A.I.R. are described in (Vara et al., 2019). To assign differential gene expression between samples we used the DESeq algorithm as implemented in the A.I.R. package. In the case of the Rbf knockdown experiment, reads were mapped and aligned using Hisat2 and StringTie. Differential expression was analysed using DESeq2. Volcano plots and heatmaps were generated using DEBrowser (Kucukural et al., 2019) in RStudio.

Gene ontology analysis was performed using the clusterProfiler package in the shinyGO web platform for both experiments (Ge et al., 2020; Yu et al., 2012). Overlapping categories were simplified using the dropGO function in the RStudio version of clusterProfiler and the 20 most significantly-enriched categories were plotted. Heatmaps were generated using the DEBrowser package for the genes contained in the highlighted GO category.

The corresponding raw datasets for the RNAseq experiments have been deposited online. Differentially expressed genes are listed in the supplementary information (Table S2).

Imaging of mitochondria

Mitochondria were imaged using TMRM (ThermoFisher Scientific #T668) or Mitotracker Red CMX Ros (Cell Signaling Technology #9082) in live samples. Flies from *tj*^{ts} crosses raised at 18°C in rescue experiments or *tj* crosses at 25°C in overexpression of Srl Delg alone were collected 0-4 days after eclosion and shifted to 29°C for 10 days. Testes were dissected in Schneider's medium and incubated in Schneider's medium with 25 nM TMRM or 50 nM Mitotracker Red for an hour.

Electron microscopy

tj^{ts} crosses were kept at 18°C. Flies were collected 0-4 days after eclosion and shifted to 29°C for 10 days. Testes were dissected and fixed in 1% glutaraldehyde/paraformaldehyde for 1.5 hours. Samples were then embedded in paraffin and sectioned for imaging.

QUANTIFICATION AND STATISTICAL ANALYSIS

For CySC counts, all Zfh1-positive and Eya-negative cells were counted. For GFP and TMRM intensity quantifications, control and experimental flies were dissected on the same day and processed simultaneously. Processing of images for the analysis of mitochondrial morphology was carried out in Image J by using the 'subtract background' and 'despeckle' tools, after which a fast Fourier transform bandpass filter was applied, modifying a previously described pipeline (Merrill et al., 2017). The circularity index of individual mitochondria was measured using the 'analyze particles' function.

Statistical tests were carried out using GraphPad Prism. For comparison of clone recovery rates, Fisher's exact test was used, for all other experiments, the test is indicated in the main text and figure legend. Numbers shown are mean ± SEM.

Cell Reports, Volume *39*

Supplemental information

Cell-cycle exit and stem cell differentiation are coupled through regulation of mitochondrial activity in the *Drosophila* testis

Diego Sainz de la Maza, Silvana Hof-Michel, Lee Phillimore, Christian Bökel, and Marc Amoyel

Table S1. Clone recovery rates (% testes) for the indicated genotypes. Related to Figures 1, 2 and S2.

Clone recovery rates that are significantly different from their respective controls are indicated by blue shading. All others are not significantly different, as determined by Fisher's exact test.

N.B. the *Cdk4*^{s4639} experiment was carried out separately from other FRT^{42D} experiments with an independent control.

ND: Not determined.

Genotype	2 dpci (n)	7 dpci (n)	14 dpci (n)
Control (*FRT*^{40A})	43 (23)	48 (54)	ND
CycE^{AR95}	16 (31)	0 (37)	ND
CycE^{WX}	60.5 (43)	29.3 (75)	ND
Control (*FRT*^{42D})	67 (95)	55 (148)	65 (46)
Dp^{a3}	67 (18)	56 (55)	66 (29)
Dp^{a4}	ND	62 (47)	67 (30)
Cdk4^{k06503}	53 (38)	47 (34)	ND
Control (*FRT*^{42D})	67 (42)	50 (42)	ND
Cdk4^{s4639}	ND	33 (52)	ND
Control (*FRT*^{82B})	42 (45)	45 (93)	ND
E2f1^{rM729}	45 (33)	33 (55)	ND

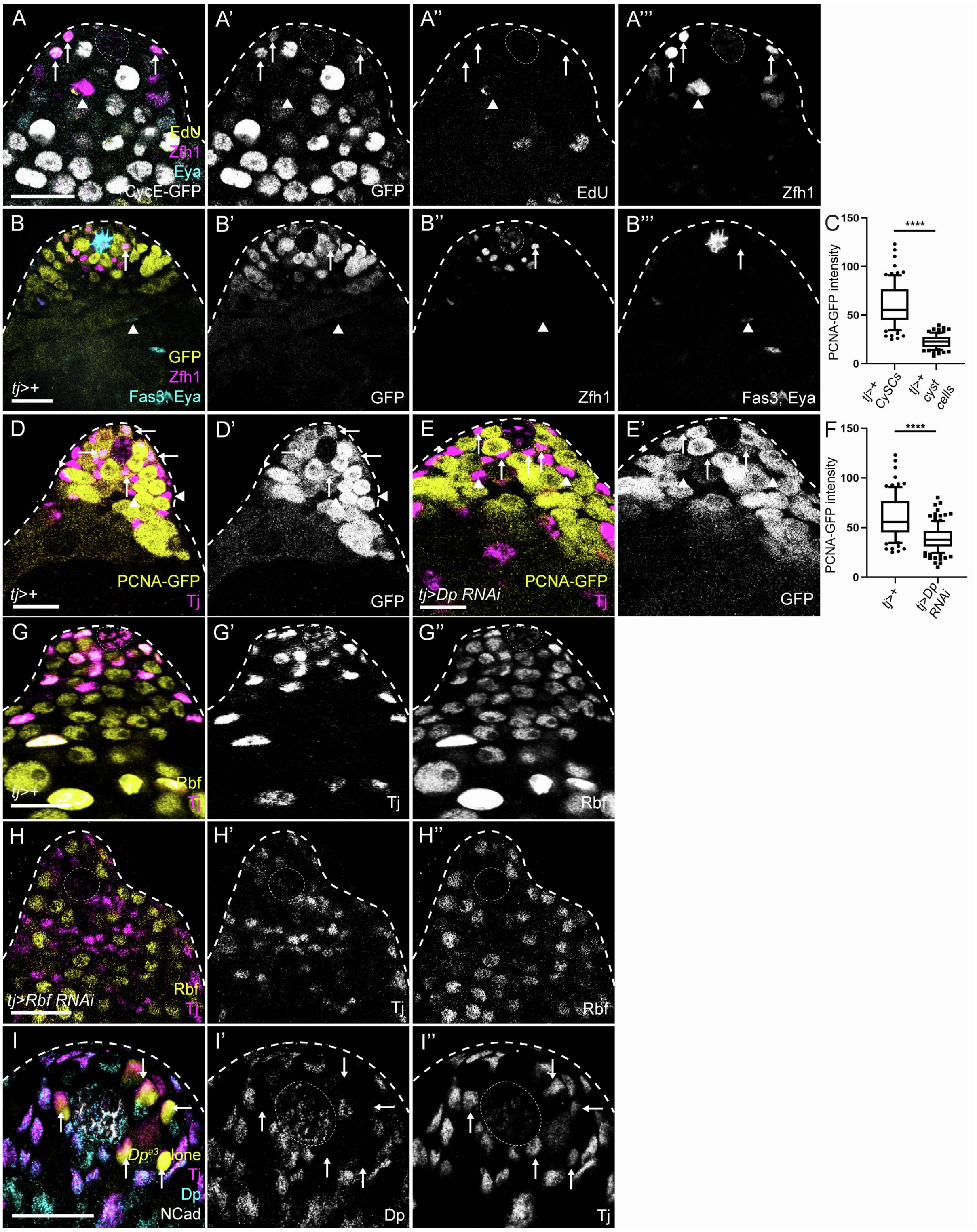

Figure S1. Expression and endogenous activity of G1/S regulators in the *Drosophila* testis. Related to Figures 1 and 2. (A) Testis from a CycE protein trap (CycE-GFP) line, labelled with EdU (yellow, A") and antibodies against GFP (white, A'), Zfh1 (magenta, A'") to label CySCs and Eya (cyan) to label cyst cells. GFP expression is detected in a subset of CySCs (arrows), but absent from CySCs that are in S-phase, identified by EdU incorporation (arrowhead). (B) Expression of the canonical E2f1/Dp reporter *PCNA-GFP* (yellow, single channel in B') in the testis. Zfh1 (magenta, B') labels CySCs and

their immediate daughters. Eya labels differentiated cyst cells (cyan, B'''). *PCNA-GFP* is present in a subset of CySCs (arrow), consistent with a cyclic pattern of expression of PCNA. No signal can be observed in Eya-positive cyst cells (arrowhead). (C) Quantification of PCNA-GFP signal in CySCs and differentiated cyst cells, showing a significant difference in GFP intensity. **** denotes $P<0.0001$, Mann-Whitney test. (D-F) Knockdown of Dp decreases PCNA-GFP signal. Tj (magenta) labels CySCs and early cyst cells. In controls (D), GFP expression is detected in Tj-positive CySCs adjacent to the hub (arrows), while no expression is present in differentiated cyst cells (arrowhead). (E) Dp knockdown in the somatic lineage results in decreased expression in Tj-positive cells around the hub (arrows), indicating that PCNA-GFP expression reports on endogenous E2f1/Dp activity. (F) Quantification of PCNA-GFP intensity in CySCs adjacent to the hub in controls and Dp knockdowns. **** denotes $P<0.0001$, Mann-Whitney test. (G-H) Testes labelled with antibodies against Rbf (yellow, single channels in G",H") and Tj (magenta, single channels in G',H'). In controls (G), Rbf is detected in all cells in the Drosophila testis, including CySCs adjacent to the hub (dotted line) and differentiating cyst cells. Expression increases in cyst cells distant from the hub. Rbf is also present in germ cells and weakly in hub cells. (H) Expression of Rbf RNAi with *tj-Gal4* results in a complete lack of any detectable Rbf protein in Tj-positive cells. (I) Positively-labelled clones mutant for *Dp* labelled with antibodies against Dp (cyan, I'), Tj (magenta, I"), GFP to mark the clone (yellow) and NCad to mark the hub (white). Mutant clones lack Dp protein (arrows). Dotted lines outline the hub. Scale bars: 20μm.

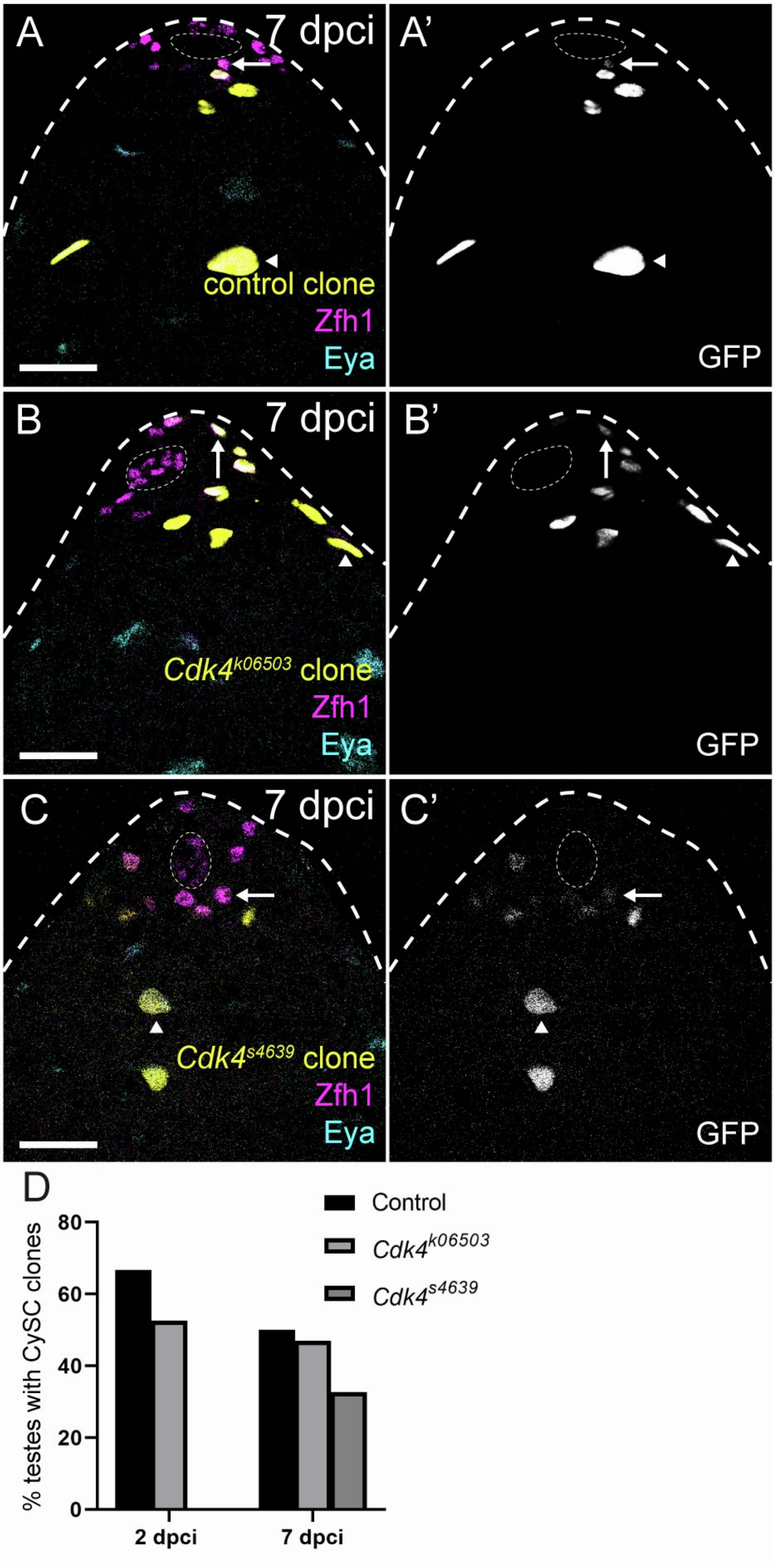

A
7 dpci
control clone
Zfh1
Eya
A'
GFP
B
7 dpci
Cdk4^{k06503} clone
Zfh1
Eya
B'
GFP
C
7 dpci
Cdk4^{s4639} clone
Zfh1
Eya
C'
GFP
D
Control
Cdk4^{k06503}
Cdk4^{s4639}
% testes with CySC clones
80
60
40
20
0
2 dpci
7 dpci

Figure S2. *Cdk4* is not required for CySC self-renewal. Related to Figure 2. Positively-marked clones labelled with GFP expression (yellow, single channels in A',B',C') were generated and recovered at 7 days post clone induction (dpci). Zfh1 (magenta) identifies CySCs and early daughters, and Eya (cyan) marks differentiated cyst cells. Control clones (A) at 7 dpci contained both Zfh1-expressing CySCs (arrow) and Eya-positive cyst cells (arrowhead). Clones mutant for independent alleles of *cdk4* (B,C) were similarly recovered at 7 dpci and contained Zfh1-expressing CySCs (arrows), indicating that *Cdk4* is not required for CySC self-renewal. (D) Quantification of clone recovery rates, showing the fraction of testes containing controls and *Cdk4* mutant CySC clones. No significant differences were observed between the recovery rates of control and mutant clones, as determined by Fisher's exact test. See Table S1 for n values. Dotted lines outline the hub. Scale bars: 20μm.

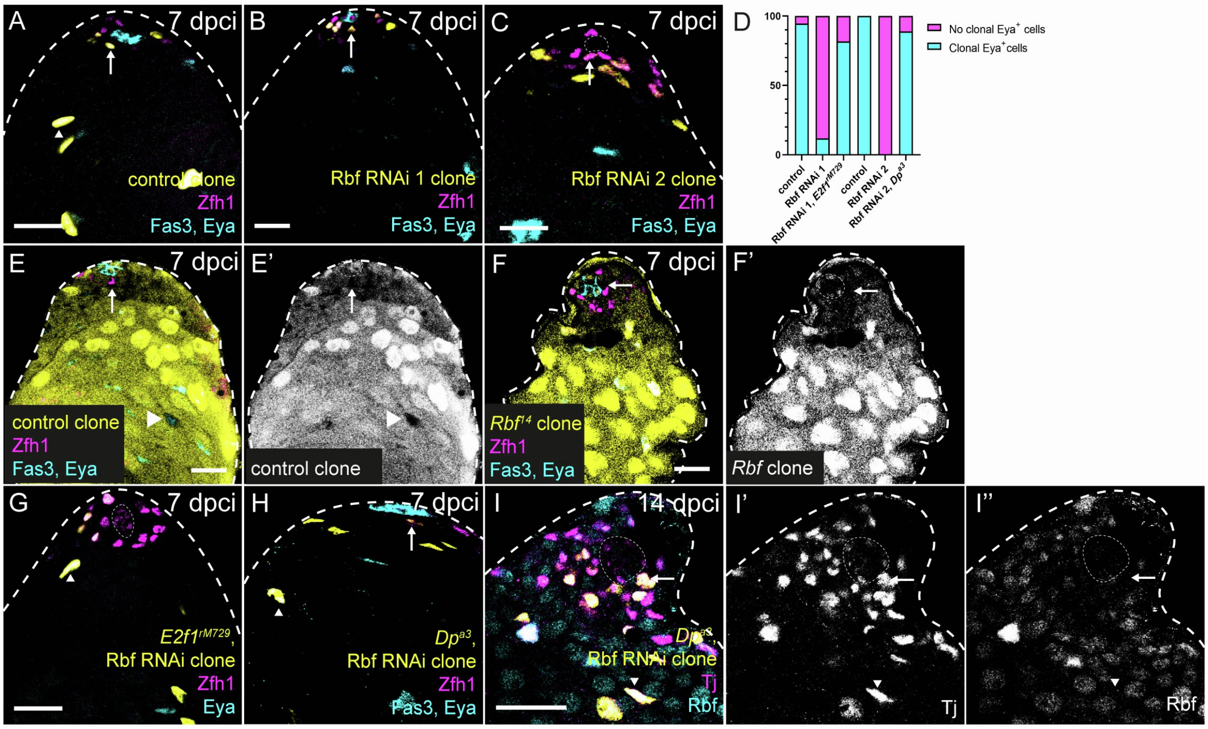

Figure S3. Clonal loss-of-function of *Rbf* recapitulates the phenotype of lineage-wide knockdown. Related to Figure 3. (A-C) Positively-marked CySC clones expressing GFP (yellow). Zfh1 (magenta) marks CySCs and early daughters, Eya (cyan) marks differentiated cyst cells and Fas3 marks the hub. Control clones at 7 dpci (A) were recovered adjacent to the hub (arrow) and contained differentiated cyst cells (arrowhead). (B,C) Clones expressing two independent RNAi constructs targeting Rbf consisted exclusively of Zfh1-expressing cells and contained no Eya-expressing cyst cells. (D) Frequency plot showing the proportion of clones containing Eya-positive cells in the different genotypes. (E,F) Control (E) or *Rbf* mutant (F) negatively-marked clones, identified by lack of GFP expression (yellow, single channel in E',F'). Zfh1 (magenta) labels CySCs, Eya (cyan) marks differentiated cyst cells and Fas3 (cyan) marks the hub. See Methods for information about genotypes. Control clones were composed of both Zfh1-positive CySCs (arrow) and Eya-positive cyst cells (arrowhead) at 7 dpci. (F) In contrast, *Rbf* hemizygous mutant clones contained only Zfh1-expressing cells (arrow), indicating that the *Rbf* mutant phenotype in CySCs is similar to RNAi knockdown. (G-I) Differentiation of Rbf RNAi clones is rescued by loss-of-function of *E2f1* or *Dp*. Clones mutant for *E2f1* (G) or *Dp* (H) in which Rbf RNAi was expressed were composed of both Zfh1-expressing CySCs (arrows) and Eya-positive differentiated cyst cells (arrowheads). (I) Rbf protein (cyan, single channel in I") is absent from *Dp* mutant clones expressing Rbf RNAi (yellow). Cyst lineage clones are identified by GFP expression and Tj expression (magenta, single channel I'). Dotted lines outline the hub. Scale bars: 20μm.

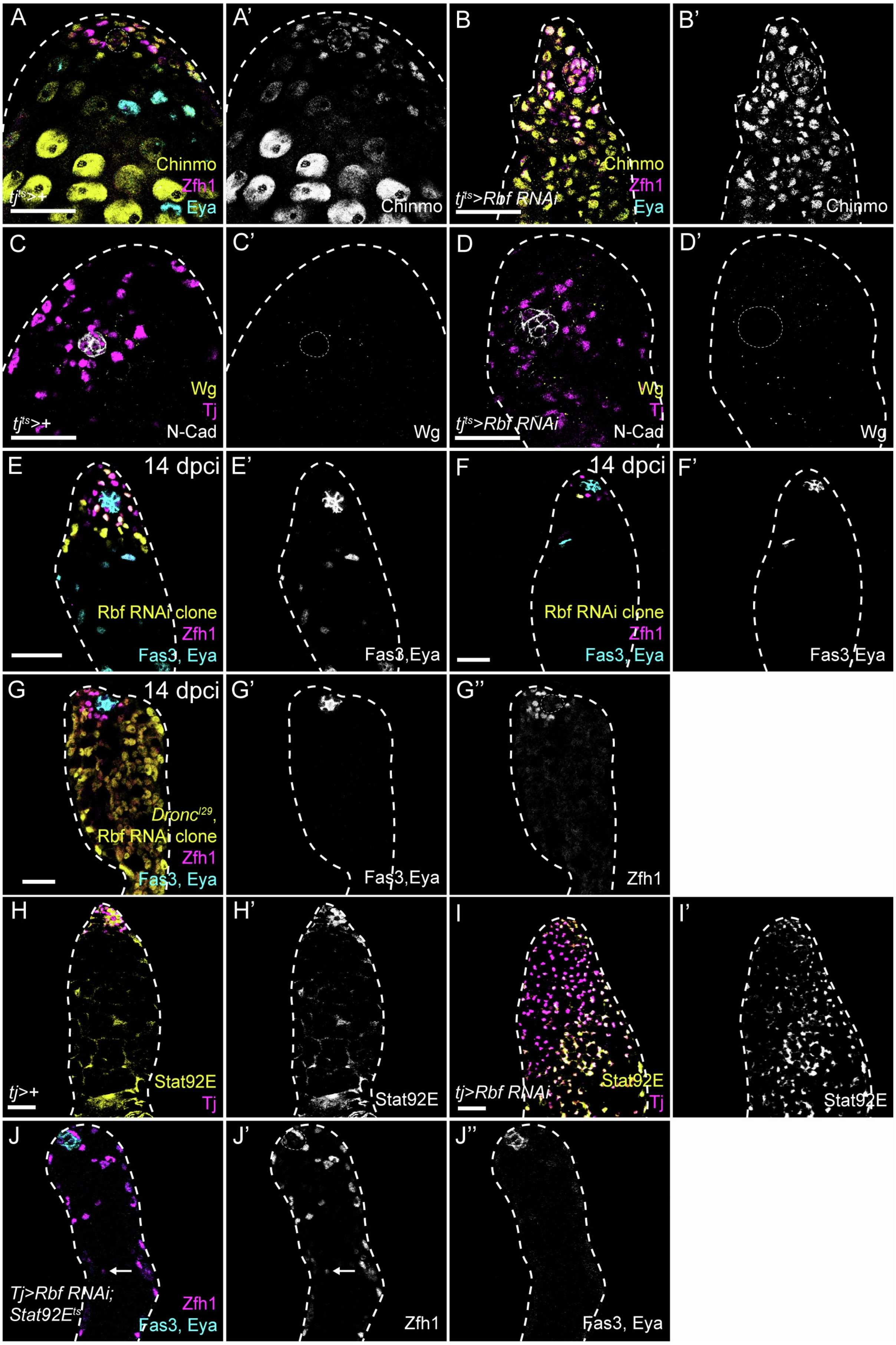

A
A'
B
B'
Chinmo
Zfh1
Eya
tj^ts>+
Chinmo
tj^ts>Rbf RNAi
C
C'
D
D'
Wg
Tj
N-Cad
E
E'
F
F'
14 dpci
Rbf RNAi clone
Fas3, Eya
Fas3,Eya
G
G'
G''
Dronc^I29,
H
H'
I
I'
Stat92E
tj>+
tj>Rbf RNAi
J
J'
J''
Tj>Rbf RNAi;
Stat92E^ts

Figure S4. Characterisation of ectopic CySCs lacking Rbf. Related to Figure 3. (A-D) Rbf-deficient cells express markers of CySCs. (A,B) Testes labelled with antibodies against Chinmo (yellow, single channel in A’,B’), Zfh1 (magenta) to mark CySCs and early daughters, and Eya (cyan), to mark differentiated cyst cells. In controls (A), Chinmo is expressed in the hub, Zfh1-positive CySCs, GSCs, identified as cells adjacent to the hub negative for Zfh1 expression, and late germ cells. Expression is absent from Eya-positive cyst cells. When Rbf was knocked down in somatic cells with tj^{ts} (B), Chinmo expression was detected in Zfh1-positive somatic cells distant from the hub. Expression of Chinmo in unlabelled early germ cells was also detected throughout the testes. (C,D) Wg expression (yellow, single channels in C’,D’) in testes labelled with antibodies against N-Cadherin (N-Cad, grey) to label the hub and Tj (magenta) to label CySCs and early cyst cells. In controls (C), Wg puncta are visible around somatic cells close to the hub. (D) Wg staining was detected far from the hub in testes with somatic Rbf knockdown indicating an expansion of CySC-like cells. (E-G) CySC clones at 14 dpci positively-labelled with GFP expression. Fas3 (cyan, single channel in E’,F’,G’) labels the hub, Zfh1 (magenta) marks CySCs and early daughter cells, and Eya (cyan) marks differentiated cyst cells. Expression of Rbf RNAi in otherwise wild type clones (E,F) resulted in clones composed only of Zfh1-positive cells and lacking Eya-positive differentiated cyst cells. While most clones contained many cells (E), indicative of proliferation, occasional clones were observed that were composed of very few cells (F). (G) Rbf RNAi expression in clones mutant for the initiator caspase *Dronc* resulted in large clones but these were still devoid of Eya-expressing cyst cells, indicating that cell death restricts the over-proliferation of Rbf-deficient cells but is not responsible for their inability to differentiate. (H-J) Increased JAK/STAT signalling does not mediate the expansion of CySC-like cells caused by Rbf knockdown. (H,I) Testes labelled with antibodies against Stat92E (yellow, single channels in H’,I’), which is stabilised where JAK/STAT signalling is active, and Tj (magenta) to mark CySCs and early cyst cells. In controls (H), Stat92E is detected around the hub. In testes somatically depleted for Rbf (I), stabilised Stat92E is present in Tj-positive cells throughout the testes. (J) Rbf knockdown in the somatic lineage in a temperature-sensitive *Stat92E* mutant ($Stat92E^{ts}$) raised for 10 days at the restrictive temperature results in expansion of Zfh1 (magenta, single channel in J’) away from the hub (arrow) and a lack of Eya-positive cells (cyan, single channel in J”), similar to Rbf knockdown in control animals. Dotted lines outline the hub. Scale bars: 20μm.

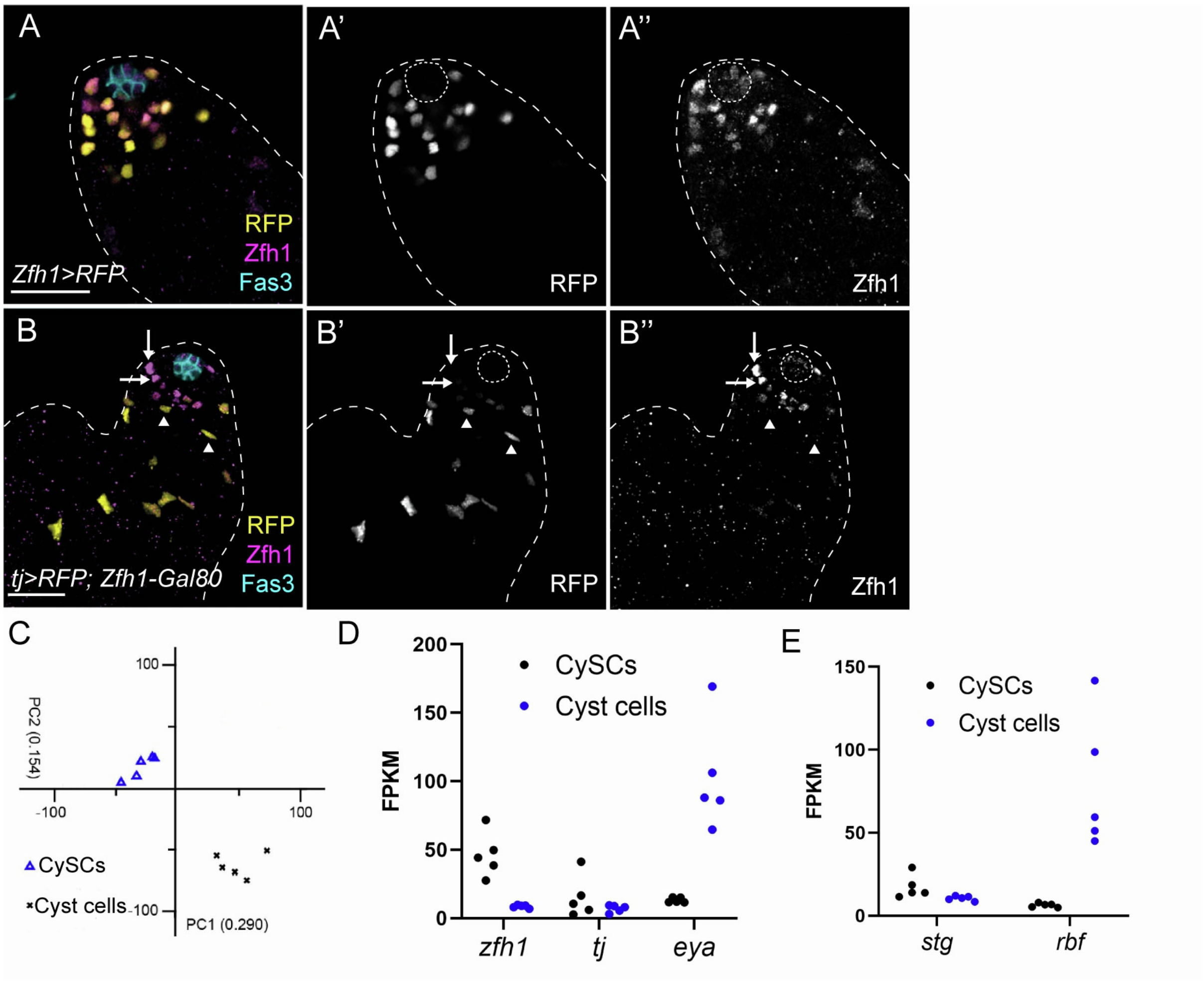

Figure S5. Effective isolation and transcriptome determination of CySCs and cyst cells. Related to Figure 6. (A) Expression of RFP (yellow, single channel A’) in *Zfh1>RFP* testes to isolate CySCs. Presence of RFP correlates with Zfh1 expression (magenta, single channel A”). The hub is labelled with Fas3 (cyan). (B) Expression of RFP (yellow, single channel in B’) in *tj>RFP; Zfh1-Gal80* testes to isolate differentiating cyst cells. Zfh1-positive cells (magenta, single channel in B”) close to the hub (Fas3, cyan) do not express RFP (arrows), whereas early differentiating cells (arrowheads) negative for Zfh1 express RFP. Dotted lines outline the hub. Scale bars: 20μm. (C) Principal component analysis of the transcriptomes of five biological replicates of sorted cell populations showing that CySCs and cyst cells cluster separately. (D) Individual values for reads (in Fragments Per Kilobase of transcript per Million mapped reads (FPKM)) for each replicate of genes encoding known markers of CySCs and cyst cells in the sorted CySC population (black) and cyst cells (blue). As expected, CySCs express higher levels of *zfh1* and *tj*, whereas early cyst cells express higher levels of *eya*. (E) Individual values for reads in FPKM of genes encoding cell cycle components. *Stg* expression which was enriched in CySCs (black) relative to cyst cells (blue), while *Rbf* was upregulated in cyst cells.

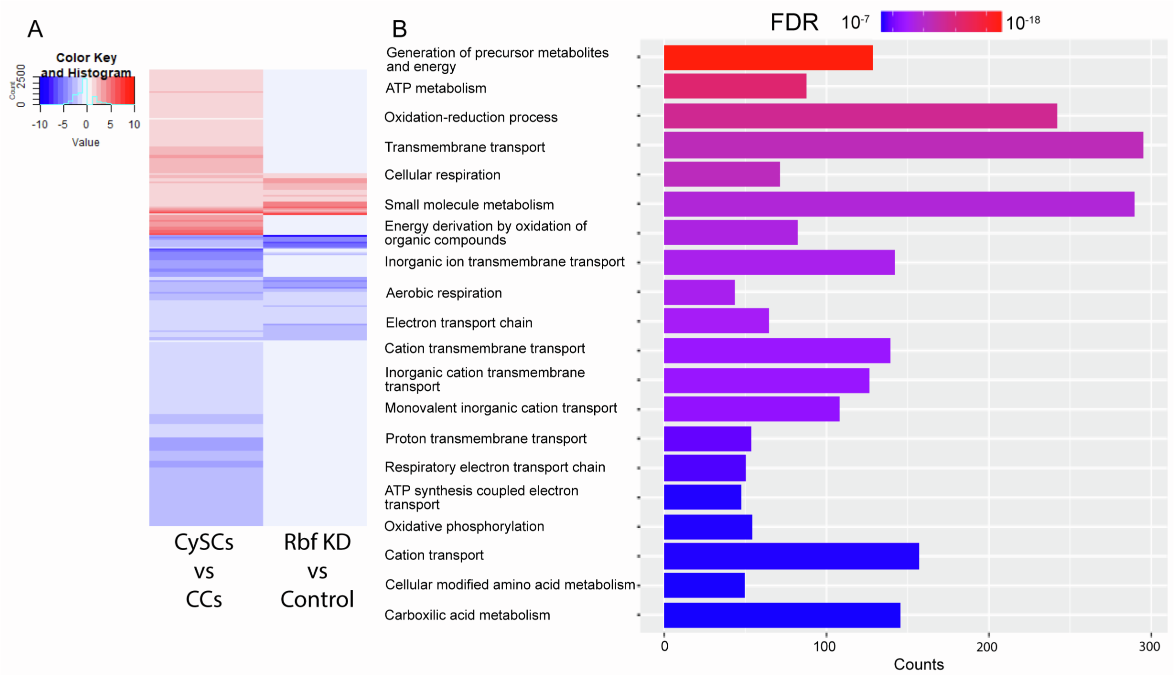

Figure S6. Overlap between the enriched transcripts in CySCs relative to cyst cells and Rbf-deficient CySCs relative to control. Related to Figure 6. (A) Heatmap representing the shared gene differential expression among experiments. Differentially-expressed genes between CySCs and cyst cells (left column) were used as a reference. Genes more highly expressed in CySCs relative to cyst cells are shown in red and genes enriched in cyst cells are shown in blue. Changes for these genes in Rbf knockdown compared to controls are shown in the right column and colour-coded according to differential expression in Rbf knockdowns. Pale blue in the right column indicates genes differentially expressed between control CySCs and cyst cells that were not differentially expressed when Rbf-knockdown somatic cells were compared to controls. Approximately 27% of genes are differentially expressed in both experiments, and show similar valence of expression change. (B) Gene Ontology analysis of genes downregulated in both control CySCs compared to cyst cells and Rbf-deficient cells compared to control. The x axis represents the number of genes in each biological process. Colours of the bars represent the FDR of each category. Shared biological processes include oxidation-reduction processes and many categories related to ATP metabolism, suggesting that somatic knockdown of Rbf results in a similar metabolic gene expression profile to CySCs.

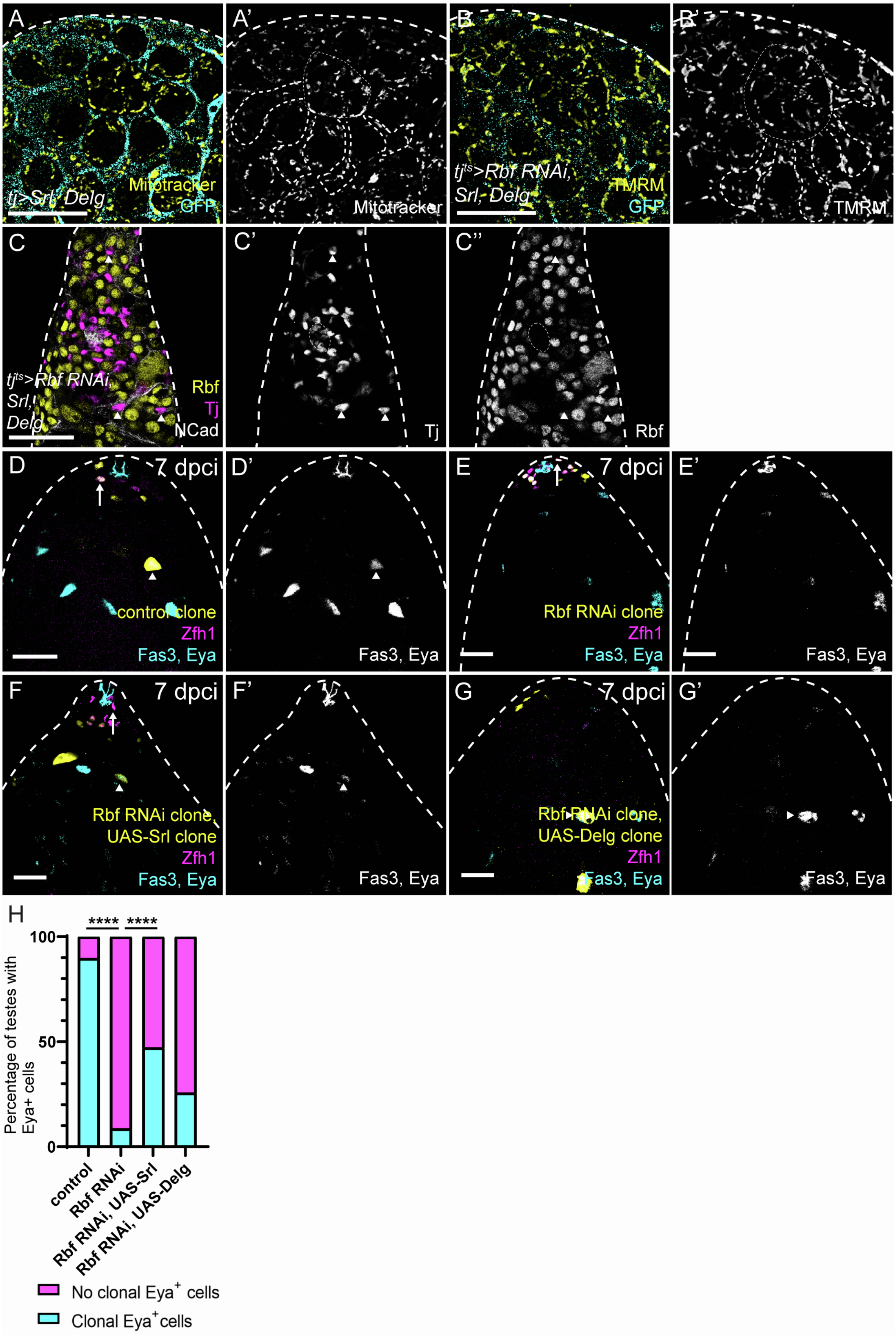
A
tj>Srl, Delg
Mitotracker
GFP
A'
Mitotracker
B
tj^ts>Rbf RNAi, Srl, Delg
TMRM
GFP
B'
TMRM
C
tj^ts>Rbf RNAi, Srl, Delg
Rbf
Tj
NCad
C'
Tj
C''
Rbf
D
7 dpci
control clone
Zfh1
Fas3, Eya
D'
Fas3, Eya
E
7 dpci
Rbf RNAi clone
Zfh1
Fas3, Eya
E'
Fas3, Eya
F
7 dpci
Rbf RNAi clone, UAS-Srl clone
Zfh1
Fas3, Eya
F'
Fas3, Eya
G
7 dpci
Rbf RNAi clone, UAS-Delg clone
Zfh1
Fas3, Eya
G'
Fas3, Eya
H
****
****
100
50
0
Percentage of testes with Eya+ cells
control
Rbf RNAi
Rbf RNAi, UAS-Srl
Rbf RNAi, UAS-Delg
No clonal Eya+ cells
Clonal Eya+ cells

Figure S7. Overexpression of Srl and Delg increases mitochondrial activity and rescues differentiation in Rbf knockdown testes. Related to Figure 7. (A) Testes co-expressing GFP (cyan) in the cyst lineage together with Srl and Delg, labelled with the active mitochondrial dye Mitotracker Red (yellow, single channel in A') and imaged live. Individual CySCs are outlined with a dashed line in A' and the hub is outlined with a dotted line. (B) Testes co-expressing GFP (cyan) in the cyst lineage together with Rbf RNAi, Srl and Delg, labelled with the active mitochondrial dye TMRM (yellow, single channel in B') and imaged live. Individual CySCs are outlined with a dashed line in B' and the hub is outlined with a dotted line. (C) Rbf protein (yellow, C'') is absent in the somatic lineage in testes expressing Rbf RNAi together with Srl and Delg, even in somatic cells away from the hub (arrowheads), indicating that any rescue of differentiation in this background is not due to incomplete knockdown. (D-H) Differentiation is partly rescued by co-expression of Srl or Delg upon clonal loss of Rbf. (D-G) Positively-labelled clones marked by GFP expression (yellow). Zfh1 (magenta) marks CySCs and early daughters, while Eya (cyan, single channel in D',E',F',G') marks differentiated cyst cells. The hub is labelled with Fas3 (cyan). Control clones (D) contained both Zfh1-positive CySCs (arrow) and Eya-positive cyst cells (arrowhead), while only Zfh1-expressing cells were detected in Rbf RNAi-expressing clones (E, arrow). Co-expression of either Srl (F) or Delg (G) with Rbf RNAi resulted in clones that contained Eya-positive cells (arrowheads). In G, the hub is in an adjacent plane. (H) Graph showing the frequency of clones containing Eya-positive cells (cyan bars). Only 9% of Rbf RNAi-expressing clones contained Eya-positive cells (n=49 for control, n=46 for Rbf RNAi, $P<0.0001$ compared to control clones, Fisher's exact test). Co-expression of Srl resulted in a significant rescue compared to Rbf RNAi alone to 47% (n=36, $P<0.0001$), while 26% of clones co-expressing Delg contained Eya-positive cells, approaching statistical significance (n=35, P=0.06). Dotted lines outline the hub. Scale bars: 20μm.
